# Molecular evidence of the avocado defense response to *Fusarium kuroshium* infection: a deep transcriptome analysis using RNA-Seq

**DOI:** 10.7717/peerj.11215

**Published:** 2021-04-14

**Authors:** Claudia-Anahí Pérez-Torres, Enrique Ibarra-Laclette, Eric-Edmundo Hernández-Domínguez, Benjamín Rodríguez-Haas, Alan-Josué Pérez-Lira, Emanuel Villafán, Alexandro Alonso-Sánchez, Clemente de Jesús García-Ávila, José-Abrahán Ramírez-Pool, Diana Sánchez-Rangel

**Affiliations:** 1 Catedrático CONACyT en la Red de Estudios Moleculares Avanzados, Instituto de Ecología A.C., Xalapa, Veracruz, México; 2 Red de Estudios Moleculares Avanzados, Instituto de Ecología A.C., Xalapa, Veracruz, México; 3 Centro Nacional de Referencia Fitosanitaria, Servicio Nacional de Sanidad, Inocuidad y Calidad Agroalimentaria, Tecámac, Estado de México, México; 4 Departamento de Biotecnología y Bioingeniería, Centro de Investigación y de Estudios Avanzados del Instituto Politécnico Nacional, Ciudad de México, México

**Keywords:** *Fusarium kuroshium*, Avocado stems, *Fusarium* dieback, Fungal alcohol metabolism, Nucleotide-binding leucine-rich receptors (NLRs)

## Abstract

*Fusarium kuroshium* is a novel member of the Ambrosia *Fusarium* Clade (AFC) that has been recognized as one of the symbionts of the invasive Kuroshio shot hole borer, an Asian ambrosia beetle. This complex is considered the causal agent of *Fusarium* dieback, a disease that has severely threatened natural forests, landscape trees, and avocado orchards in the last 8 years. Despite the interest in this species, the molecular responses of both the host and *F. kuroshium* during the infection process and disease establishment remain unknown. In this work, we established an in vitro pathosystem using Hass avocado stems inoculated with *F. kuroshium* to investigate differential gene expression at 1, 4, 7 and 14 days post-inoculation. RNA-seq technology allowed us to obtain data from both the plant and the fungus, and the sequences obtained from both organisms were analyzed independently. The pathosystem established was able to mimic *Fusarium* dieback symptoms, such as carbohydrate exudation, necrosis, and vascular tissue discoloration. The results provide interesting evidence regarding the genes that may play roles in the avocado defense response to *Fusarium* dieback disease. The avocado data set comprised a coding sequence collection of 51,379 UniGenes, from which 2,403 (4.67%) were identified as differentially expressed. The global expression analysis showed that *F. kuroshium* responsive UniGenes can be clustered into six groups according to their expression profiles. The biologically relevant functional categories that were identified included photosynthesis as well as responses to stress, hormones, abscisic acid, and water deprivation. Additionally, processes such as oxidation-reduction, organization and biogenesis of the cell wall and polysaccharide metabolism were detected. Moreover, we identified orthologues of nucleotide-binding leucine-rich receptors, and their possible action mode was analyzed. In *F. kuroshium*, we identified 57 differentially expressed genes. Interestingly, the alcohol metabolic process biological category had the highest number of upregulated genes, and the enzyme group in this category may play an important role in the mechanisms of secondary metabolite detoxification. Hydrolytic enzymes, such as endoglucanases and a pectate lyase, were also identified, as well as some proteases. In conclusion, our research was conducted mainly to explain how the vascular tissue of a recognized host of the ambrosia complex responds during *F. kuroshium* infection since *Fusarium* dieback is an ambrosia beetle-vectored disease and many variables facilitate its establishment.

## Introduction

Phytopathogens in the *Fusarium* genus are the causal agents of several diseases in numerous hosts that are important in agriculture and forestry. The symptoms of these diseases include wilting, necrosis, dieback and vascular staining (Ma et al., 2013). They are necrotrophic due to their capacity to produce and secrete both toxins and lytic enzymes that facilitate disease establishment and cell death (Ma et al., 2013). Several pathogenetic mechanisms in different *Fusarium* species as well as different host molecular responses during specific interactions have been characterized, i.e., those in *Fusarium oxysporum*-tomato, *Fusarium verticillioides*-maize, and *Fusarium graminearum*-wheat (Rauwane et al., 2020; Srinivas et al., 2019; Wang et al., 2016); however, new *Fusarium* species continually emerge and require attention, especially if they are responsible for important phytosanitary problems. This is the case for *Fusarium kuroshium*, an ambrosia fungus that forms an obligate symbiosis with the Kuroshio shot hole borer (KSHB), an ambrosia beetle that was recently formally named *Euwallacea kuroshio* Gomez and Hulcr (Gomez et al., 2018; Smith et al., 2019). This beetle is an invasive pest that is responsible for *Fusarium* dieback (FD) in California, USA, particularly in San Diego county (Na et al., 2017). As an ambrosia beetle, KSHB constructs galleries in the xylem of host trees where they cultivate their symbiotic fungi for nutrition purposes; simultaneously, the fungi are dispersed and inoculated into the plant host (Freeman et al., 2016; Hulcr & Stelinski, 2017; Six & Wingfield, 2011). Eskalen and colleagues recently reported KSHB-FD as a pest-disease complex with two fungal mutualists: *F. kuroshium* and *Graphium kuroshium* (Na et al., 2017). The host range of KSHB includes economically important crops such as avocado (*Persea americana*) and important forest species such as *Populus nigra* and *Salix lasiolepis*. In 2016, the presence of KSHB was reported for the first time in Tijuana, Baja California, Mexico (García-Avila et al., 2016), and KSHB is currently classified as a quarantine pest (SENASICA, 2019).

Because of the importance of this complex, studies on *(i)* the identification of both beetle and pathogen fungi for phylogenetic classification (Gomez et al., 2018; Smith et al., 2019; Na et al., 2017), *(ii)* the confirmation of *F. kuroshium* and *G. kuroshium* as causative agents of FD by proving Koch’s postulates (Na et al., 2017) and *(iii)* the development of molecular tools for ambrosia fungi identification (Carrillo et al., 2019; Vázquez-Rosas-Landa et al., 2021) have been conducted to provide assertive diagnostic strategies. However, only one study exists that attempts to comprehensively understand the pathogenetic mechanisms of the fungus at the molecular level (Sanchez-Rangel et al., 2018). In that study, the ability of *F. kuroshium* (previously named *Fusarium sp*. associated with KSHB) to grow at different pH levels was evaluated, and the associated transcriptomic responses were analyzed. The study revealed that this fungus can induce gene transcription related to virulence, such as proteases, PKs, and ABC transporters, at both alkaline and acidic pH. However, no data have been generated regarding the molecular responses of the host.

However, the generation of transcriptomic data, for example, by RNA-seq, has proven to be a useful tool in the study of plant-microbe interactions mainly for *(i)* analyzing host responses to identify genes involved in disease resistance (Kebede et al., 2018), *(ii)* investigating the differences between resistant and susceptible host genotypes (Li et al., 2018; Pan et al., 2018; Silvia Sebastiani et al., 2017), and *(iii)* studying a specific group of genes that play important roles during an interaction (Sahu et al., 2017; Wang et al., 2018). Transcriptomic data also become very relevant when an assembled transcriptome of the host during infection is revealed for the first time (Lin et al., 2017; Visser et al., 2018). In this context, we designed a reproducible in vitro pathosystem with avocado stems (*P. americana* var. Hass) artificially infected with *F. kuroshium* and analyzed the transcriptomic response of both organisms to identify biologically relevant functional categories.

Recently, the avocado genome was sequenced, and the available draft in the public databases consists of 912.6 Mb and contains approximately 24,616 protein-coding genes, which represent 85% completeness according to the single-copy orthologue genes conserved among the Embryophyta clade (Rendon-Anaya et al., 2019). However, even though a reference genome is available, we employed a de novo assembled transcriptome assembly approach in the current study to identify transcripts that likely have been missed by the genome assembly process or are just not appropriately annotated, as well as to identify genes involved in the underlying mechanism of *F. kuroshium* infection in avocado.

This is the first study to provide molecular data related to FD, a currently emergent phytosanitary problem in California, USA, and Tijuana, Baja California, Mexico, thus contributing to the understanding of this disease to aid research on the development of disease management strategies.

## Materials and Methods

### Strain, media, and culture conditions

The *F. kuroshium* HFEW-16-IV-019 strain (first identified as *F. euwallaceae* and later as *Fusarium spp*. associated with KSHB) (Ibarra-Laclette et al., 2017; Sanchez-Rangel et al., 2018) was provided by the Centro Nacional de Referencia Fitosanitaria (CNRF). All experiments were conducted following biosecurity protocols. Conidia were stored in 25% glycerol at −80 °C and propagated on potato dextrose agar (PDA) when necessary, and the plates were incubated for 5–7 days at 28 °C in darkness. Fungal spores were collected by gently shaking the plate with 3–5 mL of sterile water at room temperature. The suspension was collected and transferred to 2-mL Eppendorf tubes and centrifuged at 13,000 rpm for 15 min. The supernatant was decanted, and the conidia were washed twice with sterile water. Total conidia were counted under an optical microscope in a hemocytometer, and a suspension containing 1 × 10^8^ conidia/mL was prepared in sterile water.

### Avocado stem inoculation

One-year-old healthy avocado trees (*P. americana* cv. Hass) were acquired from the Salas certified plant nursery at Uruapan, Michoacán, Mexico. The avocado trees were acclimated for 4–6 months in a greenhouse until used. Selected branches of approximately 30–40 cm in length and 1 cm in diameter were cut with garden scissors and cleaned with sterile water, and each stem was then cut into fragments of 3.5–4 cm in length. A hole was drilled into the center of each avocado stem with a 1/16″ Dremel^®^. The injured stems were placed into humid chambers prepared by placing sterile filter paper discs in the bottom of Petri dishes (150 × 20 mm) and adding 4–5 mL of sterile distilled water. Each stem was inoculated with 40 µL of conidial suspension (1 × 10^8^ conidia/mL) or H_2_O for the control. The humid chambers were maintained at 27 °C and 16 h light/8 h dark for 1, 4, 7 and 14 days post-inoculation (dpi).

### RNA isolation, library preparation, and sequencing

Infected and uninfected stems (at 1, 4, 7 and 14 dpi) were frozen with liquid nitrogen and then pulverized using a mortar and pestle. Total RNA was isolated from each sample using PureLink^®^\ Plant RNA Reagent (Thermo Fisher Scientific, Waltham, MA, USA) following the manufacturer’s protocol. RNA concentration was measured on a NanoDrop 2000c spectrophotometer (Thermo Scientific, Thermo Fisher Scientific, Waltham, MA, USA), and RNA integrity (RIN ≥ 8.5) was evaluated on an Agilent 2100 Bioanalyzer using an RNA 6000 Nanokit (Agilent Technologies, Santa Clara, CA, USA). Then, 200 ng of total RNA from each selected sample was used to generate the corresponding sequencing libraries. We used the RNA-seq method based on poly(A) selection, which enriches eukaryotic mRNA and other polyadenylated RNA molecules. Three independent biological replicates from each selected sample were processed. Using the TruSeq RNA Sample Preparation Kit (Illumina, San Diego, CA, USA) and index codes to identify each sample independently, RNA sequencing libraries were generated in the Massive Sequencing Unit of the Ecology Institute (INECOL, Veracruz, México) located in Xalapa, Veracruz, Mexico, using Illumina sequencing instrumentation (NextSeq500). Multiplexed libraries were sequenced on a single flow cell of the NexSeq500 platform (Illumina, San Diego, CA, USA) to generate 150 bp paired-end reads. The RNA-seq data have been deposited in the Short Read Archive (SRA) of the National Center for Biotechnology Information (NCBI) with accession number PRJNA655460.

### Assembly and identification of protein-coding regions in RNA transcripts

Before the assembly process, low-quality reads (those with an average Phred score of less than 30 and in which only 10% of the bases that belonged to the reads had a Phred score below the quality permitted (20)) were removed with a Phyton-based script (https://github.com/Czh3/NGSTools/blob/master/qualityControl.py). Prior to de novo assembly, screening was performed to identify fungus-like sequences using the Hisat2 v2.1.0 program (Kim, Langmead & Salzberg, 2015). The sequences used as references were a unique transcript collection (Sanchez-Rangel et al., 2018) and the complete genome sequence (Ibarra-Laclette et al., 2017) available in the GenBank public database.

High-quality (HQ) reads were divided into two data sets of avocado sequences and fungus-like sequences. HQ paired-end reads obtained from the libraries of the uninfected samples plus sequences from infected samples that did not match *F. kuroshium* were combined and assembled using Trinity v2.4.0 software (Grabherr et al., 2011) to produce contigs (UniGenes). The UniGenes were assayed with SeqClean software (https://sourceforge.net/projects/seqclean/files/) to remove poly A/T tails, ends rich in Ns (undetermined bases) and/or low-complexity regions from the sequences. Only the resulting UniGenes with a minimum length of 200 bp were selected and processed with AlignWise (Evans & Loose, 2015). After correcting the frameshifts, a data set for the avocado transcriptome with coding sequences (CDS) and their corresponding proteins was created. Redundant sequences were eliminated using the BlastClust algorithm (https://www.ncbi.nlm.nih.gov/Web/Newsltr/Spring04/blastlab.html). A CDS was considered redundant if it showed an identity of at least 95% over 90% or more of the length of the sequences being compared. Only CDSs that, once translated, generated peptides or proteins longer than 75 nucleotides were retained and used for further analyses.

### Functional annotation of avocado UniGenes

To annotate the avocado transcriptome, we searched homologous protein sequences and performed a functional classification based on Gene Ontology (GO) terms (Ashburner et al., 2000). First, the resulting proteins translated from the assembled UniGenes were compared with a reference database using the BLASTp algorithm (*e*-value 10^−5^) (Altschul et al., 1990). The list of plant species used as references includes representative, well-annotated species that were recently used (Rendon-Anaya et al., 2019) to inform ancient evolutionary relationships between avocado and some of the major land plant lineages eudicotyledons: *Amborella trichocarpa* (basal angiosperms), *Solanum lycopersicum* (asterids, Solanales), *Vitis vinifera* (rosids incertae sedis, Vitales), *Prunus persica* (rosids, Rosales), and *Arabidopsis thaliana* (rosids, Brassicales); monocotyledons: *Musa acuminata* (commelinids, Zingiberales) and *Zea mays* (commelinids, Poales). In addition, the putative protein domains contained within the translated UniGenes were identified using Hidden Markov model (HMM)-based searches against the Pfam database (*e*-value 10^−2^) (Finn et al., 2016; Punta et al., 2012). A PfamScan Perl-based script was used for this purpose (https://github.com/SMRUCC/GCModeller/tree/master/src/interops/scripts/PfamScan/PfamScan).

After homolog identification, avocado UniGenes were classified according to Gene Ontology terms into at least one of the three major categories (biological processes, molecular functions, and cellular components). These GO terms were attributed to avocado UniGenes based mainly on the identified *A. thaliana* homologs (https://www.arabidopsis.org/index.jsp). Finally, the avocado UniGenes were assigned to specific metabolic pathways using the Kyoto Encyclopedia of Genes and Genomes database (KEGG; Kanehisa et al., 2016). The KEGG Automatic Annotation Server (KAAS; https://www.genome.jp/tools/kaas/) was used for this purpose with the single-directional best hit (SBH) method to assign orthologues.

Orthologous genes were identified in silico using the OrthMCL v.2.0.9 pipeline (Li, Stoeckert & Roos, 2003). Then, considering an inflation value (Enright, Van Dongen & Ouzounis, 2002) (1.5 in this study) and using the Markov cluster (MCL) algorithm (Enright, Van Dongen & Ouzounis, 2002), groups of orthologues (and paralogs) can be inferred across multiple taxa. We considered a minimum input length of 30 amino acids in all compared proteins, as well as a threshold *e*-value of 10^−10^ in the BLAST step. This stringent cutoff value was chosen to avoid false-positive results. The list of species used as references was the same as that used in the BLAST step performed during the annotation process.

### Differential expression analysis

To identify differentially expressed genes during the infection process in both avocado and *F. kuroshium*, HQ reads were sequentially mapped against a specific data set that was used as a reference. In the case of avocado, HQ reads (avocado-like sequences) from infected and uninfected stems (at 1, 4, 7 and 14 dpi) were independently mapped against UniGenes obtained from the de novo assembled transcriptome. Fungus-like HQ reads identified in infected samples (at 1, 4, 7 and 14 dpi) were independently mapped to the gene models predicted for the *F. kuroshium* genome. The aligner tool Bowtie2 v2.3.5.1 (Langmead & Salzberg, 2012) was used to map the HQ reads to the corresponding data set used as references. Subsequently, the RSEM v1.3.1 package (Li & Dewey, 2011) with default parameter settings was used to calculate, for each sample, the posterior mean estimates, 95% confidence intervals, and maximum likelihood abundance estimates or expected counts (EC) for each of the UniGenes/genes used as the reference. For the identification of differentially expressed UniGenes/genes, the Bioconductor tool with the DESeq v1.24.0 package (Anders & Huber, 2010) was used. The values from the RSEM package (EC) were input into DESeq, which models the over-dispersed Poisson count data using a negative binomial model to generate the base-mean of each UniGene/gene and of each of the samples, which were compared in pairs. DESeq also computes the “fold-change” (FC) as the ratio of the base-mean calculated in the control samples divided by the base-mean estimated from the treatment sample. The data for the uninfected stem segments in the avocado sample data set were compared against those for the infected stem segments at each of the sampling times (1, 4, 7 and 14 dpi). The FC was then log_2_ transformed [log_2_(FC)]. Upregulated genes had positive log_2_ values (≥1), while downregulated genes had negative log_2_ values (≤−1). As part of its statistical tests, DESeq provides adjusted *p-*values that are calculated from all UniGene *p-*values using the Benjamini-Hochberg adjustment procedure (Benjamini & Hochberg, 1995). In the present study, an adjusted *p-*value of ≤ 0.05 was considered the threshold for identifying differentially expressed UniGenes.

Although the steps for analyzing the avocado and *F. kuroshium* data sets were similar, comparisons of the samples in the *F. kuroshium* data set at 4, 7 or 14 dpi with those at 1 dpi were made independently. Again, both a log_2_FC value ± 1.0 and an adjusted *p*-value of ≤0.05 were the criteria for identifying genes as differentially expressed genes.

### Gene clustering analysis: hierarchical clustering and k-means

Gene expression differences and their resulting profiles over time were calculated based on FC increases or decreases at the different sampling times. The infected and uninfected samples were compared at each time point, and hierarchical clustering of the log_2_-transformed data sets was performed across the different genes (and time points) using Euclidean distance and average linkage clustering. The clustering results were represented visually as a heat map dendrogram. In addition, the log_2_(FC) values calculated for each gene and throughout the time course were also grouped by k-means analysis. A maximum of 1,000 iterations and a conservative number of clusters that allowed the inclusion of the largest number of differentially expressed genes (*n* = 6) were the options selected. Both hierarchical clustering and k-means analysis were performed using Genesis software version 1.8.1 (Sturn, Quackenbush & Trajanoski, 2002).

### Functional enrichment analysis

g:Profiler (http://biit.cs.ut.ee/gprofiler/; (Raudvere et al., 2019)), a widely used web toolset for identifying functional categories enriched in gene lists, was used to identify the enriched GO terms in the list of avocado UniGenes identified as differentially expressed in response to the interaction with *F. kuroshium*. The g:SCS method was applied to perform multiple testing correction using the *p*-adjusted values with a ≤ 0.05 threshold.

### Identification of avocado immune receptors

The coding of avocado UniGenes to pattern recognition receptors (PRRs) was performed based on their *A. thaliana* homologs (Nürnberger & Kemmerling, 2006) and the presence of the LysM motif in some of them (Bozsoki et al., 2020) was confirmed through the Pfam annotation.

To identify UniGenes that coded NLR proteins, the characteristic domains, namely, a variable N-terminal domain (TIR, CC or PWR8), an NBS domain and/or a C-terminal LRR domain, were recognized (Collier, Hamel & Moffett, 2011; Xiao et al., 2001; Zhong & Cheng, 2016; Monteiro & Nishimura, 2018). First, we identified all UniGenes that were grouped as orthologues of the well-characterized *A. thaliana* NLR receptors (Meyers et al., 2003). Then, using the information from the Pfam annotation, each of the UniGene sequences containing at least one of the previously mentioned domains was individually checked using the NCBI BLAST interface and the generated graphical summary that illustrates the domains contained in the sequences; these domains are identifiable in the Conserved Domain Database (CDD) (Marchler-Bauer et al., 2011).

Avocado NLR proteins were aligned in the SeaView v4.6.1 phylogeny package (Gouy, Guindon & Gascuel, 2010) using the MUSCLE v3.8.31 program (Edgar, 2004). Phylogenies were generated in a maximum likelihood (ML) framework using PhyML v3.0 software (Guindon et al., 2010; Guindon & Gascuel, 2003) and the following parameters: model: Le Gascuel (LG); branch support: approximate likelihood-ratio test (aLRT: (Anisimova & Gascuel, 2006)); amino acid equilibrium frequencies: model-given; invariable sites: optimized; across-site rate variations: optimized; tree searching operations: best algorithm of neighbor interchange (NNI) and subtree pruning and regrafting (SPR); and starting tree: Bio Neighbor-joining (BioNJ; (Gascuel, 1997)).

Finally, the 3D structures of avocado NLR proteins were modeled by the rigid body grouping method using the SWISS-MODEL workspace (http://swissmodel.expasy.org/; Arnold et al., 2006). The structures of the *A. thaliana* ZAR1 protein in its inactive and active forms (Protein Data Bank entries 6j5t.1.A.pdb and 6j5t.1.C.pdb, respectively) were used as templates. Once the proteins were independently modeled, all of them were superimposed and visualized using the PyMOL molecular graphics system, v 2.3.4 Schrödinger, LLC. In addition, two of these avocado NLR proteins were also modeled as part of the ZAR1-RKS1-PBL2 resistosome (PDB entry 6j5t; (Wang et al., 2019a)). To assess its accuracy, each model generated was checked by its global model quality estimation (GMQE) and quaternary structure quality estimation (QSQE) scores.

## Results

### Fungal infection and disease progression

**Figure 1:**
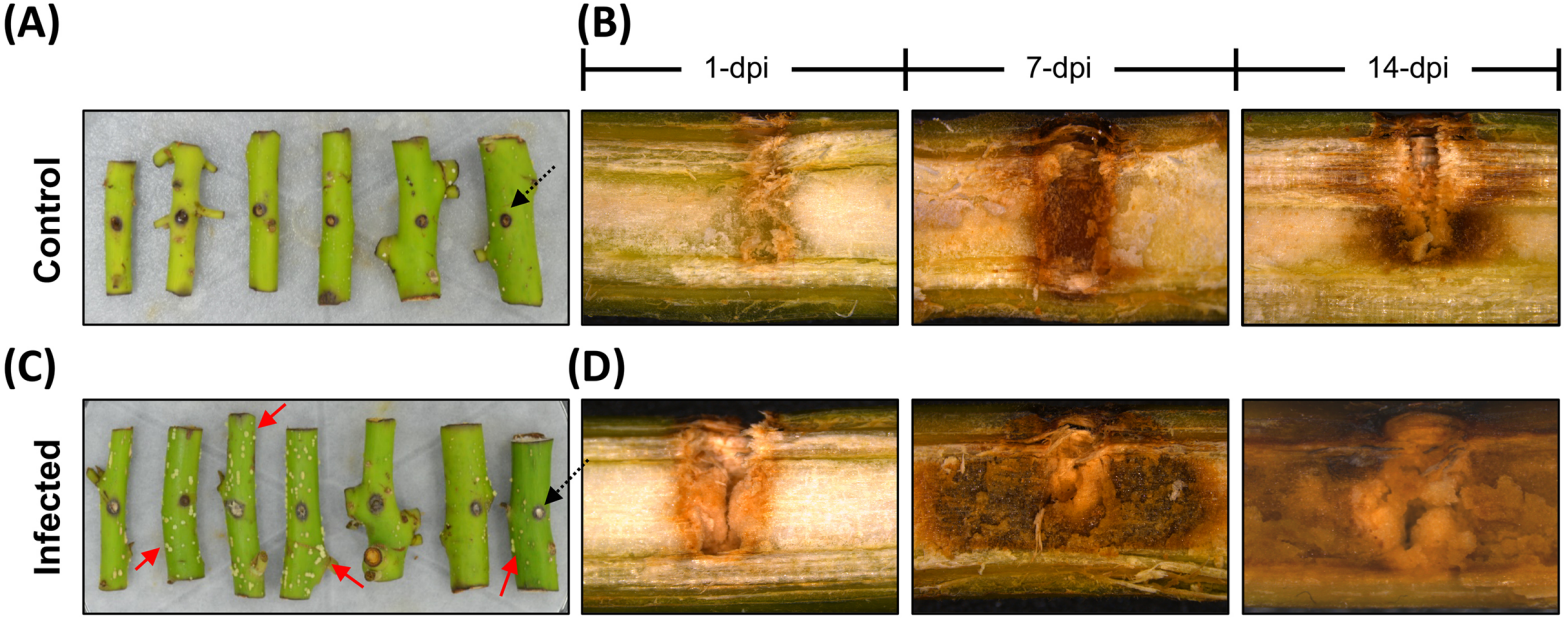
The avocado stem pathosystem. Avocado stem-segments uninfected (control), and infected with *Fusarium kuroshium* are show on images at the top and bottom (A and B, or C and D, respectively). Holes 4 mm deep (black arrowheads) were drilled in the middle of stems collected from 1-year-old avocado trees, then the stems were artificially infected with a *F. kuroshium* conidial suspension or water for the control. The infected stem segments at 7 dpi clearly show a surface exudate (red arrowheads) (C), while vascular tissue damage caused by the fungus at 1, 7 and 14 dpi (lengthwise section) is compared vs control. The images were taken with a stereoscopic microscope.

The avocado stems infected with *F. kuroshium* displayed deposits of a powdery, white-colored material along their surface after 7 dpi. This sugary exudate is recognized as a typical symptom of FD (Eskalen et al., 2013). To assess vascular damage, longitudinal cuts were made on all stems (infected and uninfected). As shown in Fig. 1, the uninfected stems displayed a response associated with the injury, showing a brownish zone around the mechanically damaged area, but the zone did not extend more than 0.2–0.4 mm, and the integrity of the vascular tissue was maintained throughout the entire stem. In contrast, the infected stems clearly showed symptoms such as brownish discoloration starting in the early infection stage (1 dpi). This symptom remained pronounced as the infection progressed, and the loss of tissue integrity was also observed at 7 and 14 dpi.

### Construction of the set of UniGenes for avocado

Of the total paired reads generated (454,682,877), approximately 30.6% were identified as poor-quality reads and were removed. In addition, 7.82% (8,516,500, 6,163,902, 5,488,581 and 15,419,814 at 1, 4, 7 and 14 dpi, respectively) were identified as *Fusarium*-like sequences and were discarded. As expected, the highest number of *Fusarium-*like sequences were identified in the samples corresponding to the late stages of infection (Fig. S1; Table S1).

After filtering out low-quality reads and removing contaminant sequences, a total of 279,667,960 paired-end reads were included in the assembly process. The same sequences were used for mapping and the differential expression analysis. As a result of the assembly process, a total of 87,343 UniGenes were generated. The corrected, nonredundant sequence data set comprised a collection of CDSs and corresponding proteins from a total of 51,379 UniGenes whose sequence lengths ranged from 75 to 15,321 bp (average length of 740.17 bp) (File S1). Only this sequence data set was considered representative of the transcriptome of *P. americana* stems and was used in the downstream analyses.

### Homolog search and functional annotation of avocado UniGenes

**Figure 2:**
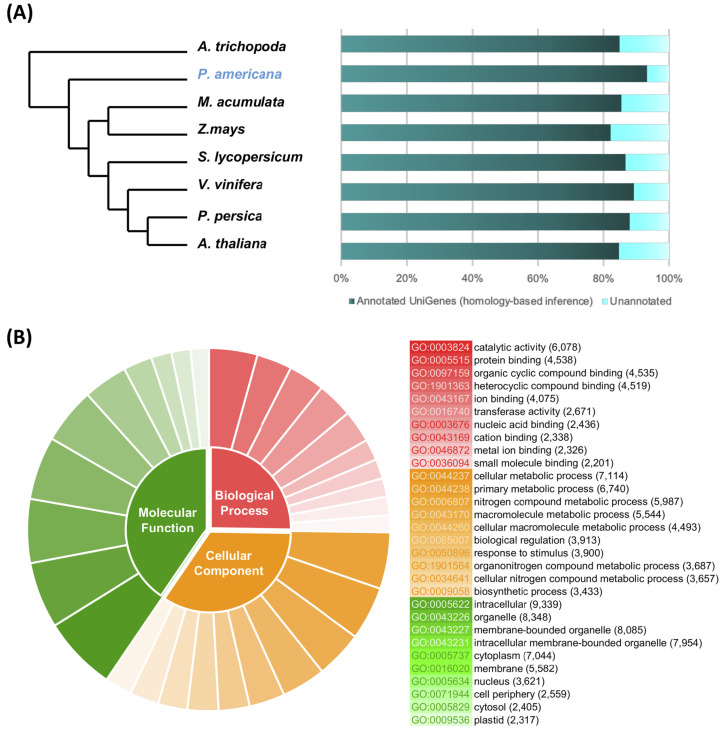
Homolog search and functional categorization of avocado UniGenes. Homolog search and functional categorization of avocado UniGenes. (A) Homologous proteins were found by BLASTp searches using the single-directional best hit (SBH) method and the predicted proteins in some species of flowering plants whose whole genome has been sequenced. These reference plant species were selected based on their phylogenetic relationships and because they represent specific lineages (Rendon-Anaya et al., 2019). The percentage of avocado UniGenes that can be annotated based on their homologous proteins varies depending on the species against which it is compared. (B) Gene Ontology functional characterization. The results are summarized and show the top ten most common GO terms for each of the three major categories (molecular functions, biological processes, and cellular components). The wedge size in the pie chart is proportional to the number of avocado UniGenes assigned to each category.

From the total number of nonredundant avocado UniGenes, 47,833 UniGenes (93.09%), showed high similarity (*e*-value ≤ 10^−5^) with proteins from at least one of the reference species (Table S2). As expected, the number of avocado UniGenes that could be annotated with respect to a reference species was distinct and depended on the quality of the predicted gene models of the reference genomes as well as their phylogenetic relationships (Fig. 2A; Table S2).

The unique accession numbers (or *loci*) that resulted from counting the homologs identified in each species that was compared with the avocado UniGenes could be considered an estimation of the minimal number of genes codified in the avocado genome or, at least, of the number of genes that represent the stem transcriptome. The number varied between 13,809 and 16,799 proteins (Table S2), depending on the species considered as a reference. According to the recently published avocado genome (Rendon-Anaya et al., 2019), this number of UniGenes represents approximately 62.17% of the total protein-coding genes identified in avocado. However, a better estimation of the completeness of the assembled transcriptome was obtained from the BUSCO v3.0.1 pipeline (Simao et al., 2015); 88.0% completeness was achieved considering a predefined set of 1,375 single-copy genes present in the Embryophyte clade.

Then, avocado UniGenes were classified and grouped into at least one of the three major GO categories. Each of the subcategories assigned was inherited based mainly on the GO annotations available (ftp://ftp.arabidopsis.org/home/tair/Ontologies/) for each *A. thaliana* protein, which were identified as homologs, and by the BLAST algorithm used in the first step of the annotation process. At least one GO term was assigned to a total of 43,493 avocado UniGenes, and the assignments included 2,393 unique GO terms from the “biological processes” category, 3,516 from “molecular functions” and 782 from “cellular components” (Table S3). Considering that more than one GO term can be assigned to a single gene, we estimated that after the annotation process, an average of four GO terms were allocated to each of the avocado UniGenes (Table S3). The most prominent GO terms in the biological process, molecular function and cellular component categories were “cellular metabolic process”, “catalytic activity”, and “intracellular”, respectively (Fig. 2B; Table S3). Finally, the avocado UniGenes were assigned to some metabolic pathways, resulting in the assignment of 3,763 unique KEGG orthology (KO) identifiers (KEGG numbers) and 2005 distinct enzyme commission numbers to a total of 20,588 avocado UniGenes (Table S4).

The last step in the annotation process was orthologue (and paralog) identification between avocado and the other angiosperm plant species used as references in this study. A total of 281,493 proteins (29,399 from *A. trichopoda*; 36,334 from *P. americana*; 44,070 from *M. acuminata*; 48,669 from *Z. mays*; 33247 from *S. lycopersicum*; 38,554 from *V. vinifera*; 30,498 from *P. persica*; and 22,474 from *A. thaliana*) were grouped in 27,751 orthogroups or OrthoMCL-defined protein families (Fig. S2; Table S5). Among them, 7,600 orthogroups were shared among all eight reference species; 257 orthogroups contained proteins from eudicotyledon plants (*S. lycopersicum*, *V. vinifera*, *P. persica*, and *A. thaliana*) but no monocots (*M. acuminate* and *Z. mays*) or proteins from ancient clades of flowering plants (*A. trichopoda* and *P. americana*) (Fig. S2; Table S5).

### Gene expression profile changes in avocado stem segments in response to infection with *F. kuroshium*

**Figure 3:**
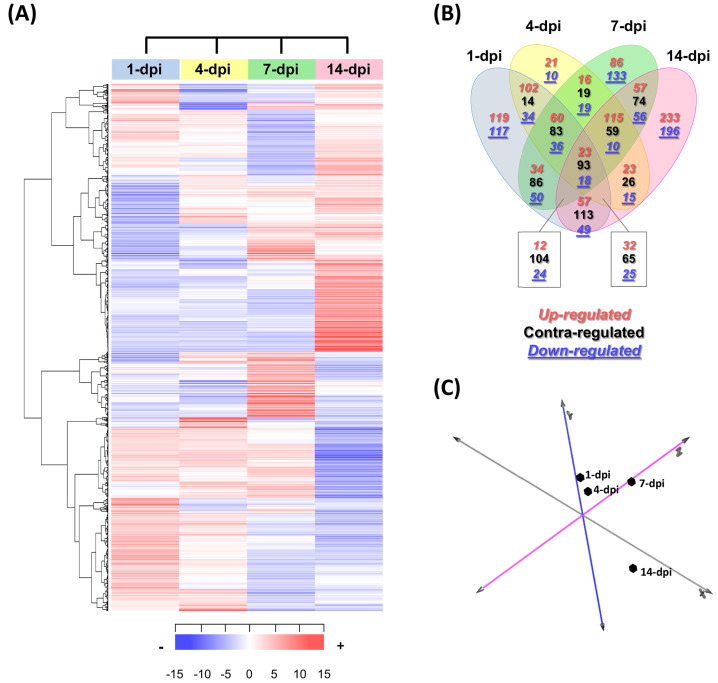
Expression profiling of the differentially expressed avocado UniGenes in response to interaction with *F. kuroshium*. (A) Heat map showing the hierarchical clustering analysis of differentially expressed genes that were identified after comparing infected vs uninfected avocado stems at 1, 4, 7, and 14 dpi. Pearson correlation distance metrics and average linkage clustering methods were used. Horizontal rows represent individual UniGenes, and vertical columns represent each sampled point. Log_2_-transformed fold-change values were used for each UniGene at each infection stage. As shown on the color scale at the bottom of the figure, blue indicates downregulation (−), red indicates upregulation (+), and white indicates unchanged expression. The Venn diagrams (B) show common or unique UniGenes identified as differentially expressed at the sampled time points. The number of genes up- or downregulated at each time point is shown using a red or blue number, respectively. (C) Principal component analysis (PCA) of the different sampling times comprising a spatiotemporal map of the avocado-*Fusarium* interaction response.

In total, 2,403 differentially expressed UniGenes were identified (fold change = 2, Log_2_FC = ±1), with an adjusted significant *p*-value of ≤ 0.05 for at least one of the sampling points (Fig. 3A; Table S6). The comparison of all the data sets indicated that the most differentially expressed UniGenes were identified at 1 and 14 dpi. At 14 dpi, a significant number of UniGenes showed significant changes in their expression profiles (Fig. 3B). As expected, principal component analysis showed the overall profile of differentially expressed UniGenes and demonstrated a clear separation between noncorrelated groups (explaining 50.4% of the variance); one group included the early stages of infection (1 and 4 dpi), and the other group represented later stages (7 and 14 dpi). There were pronounced differences between the groups, especially at 14 dpi (Fig. 3C).

Finally, to identify expression patterns throughout the infection process (1–14 dpi), we carried out a k-means clustering analysis. The preestablished number of groups to obtain was six (*n* = 6) (Fig. S3; Table S6). Cluster 1 included 310 UniGenes that showed a general trend of downregulated expression throughout the infection process, displaying a maximum level at 1 dpi and reaching a minimum level at 14 dpi. In contrast, the expression profiles of 416 UniGenes (cluster 4) showed an upregulated trend from 1 to 14 dpi, suggesting that these genes play an important role during pathogenesis. UniGenes belonging to clusters 5 and 6 (373 and 391, respectively) showed no significant changes in their expression profiles at 1, 4 and 7 dpi; however, at 14 dpi, they displayed opposite trends, with a downward trend in cluster 5 and an upward trend in cluster 6. Cluster 2 had the highest number of UniGenes (630) and displayed a slight decrease at 1 dpi, reached minimum levels at 7 dpi, and then increased at 14 dpi, returning to the expression levels displayed at 1 dpi. Finally, 284 UniGenes belonging to cluster 3 showed similar expression profiles at 1, 4 and 14 dpi, with a maximum peak of expression at 7 dpi (Fig. S3).

### Functional enrichment analysis of differentially expressed avocado genes

**Figure 4:**
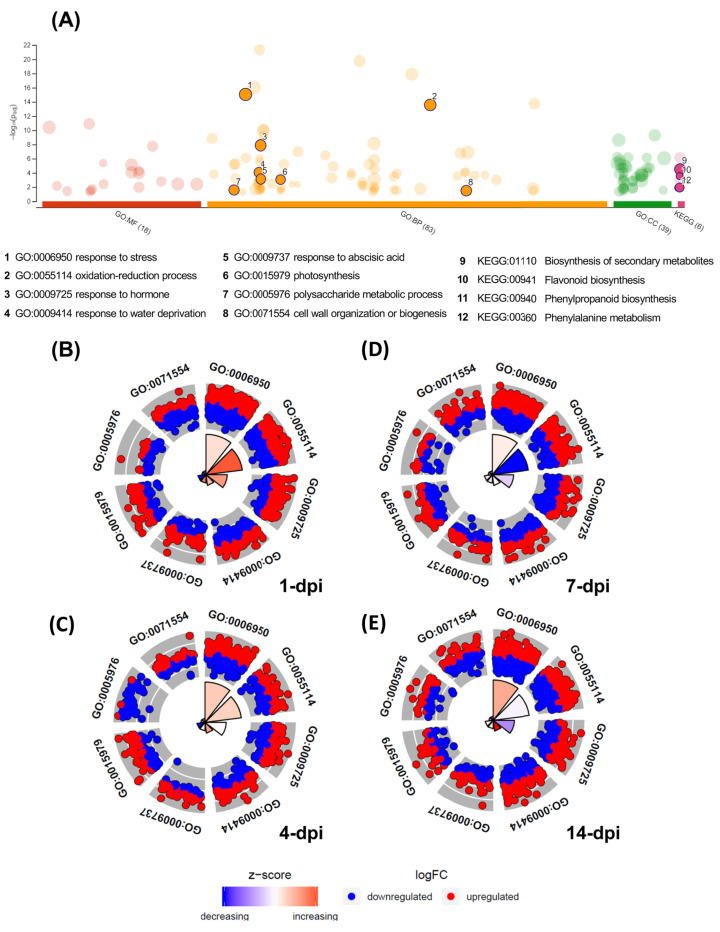
Gene Ontology enrichment of the differentially expressed avocado genes in response to interaction with *F. kuroshium*. (A) Manhattan plot illustrating the KEGG pathways and GO enrichment analysis results separated into the three major categories: MF (molecular functions), BP (biological processes), and CC (cellular components). The number in the source name in the x-axis labels shows how many GO terms or KEGG pathways were significantly enriched (g:SCS threshold, *p*-value ≤ 0.05). The circle size in the Manhattan plot corresponds to the number of genes annotated to each of the enriched categories. (B–E) Circle plot highlighting eight of the enriched biological processes considered distinctive defense responses at 1, 4, 7, and 14 dpi, respectively. Red dots are genes involved in the biological process that were upregulated; blue dots are genes that were downregulated. The height of the wedges in the inner circle section is associated with the *p*-value that was calculated to identify these categories as enriched (taller wedges are more significant, i.e., lower *p*-values), and the color of the wedges in the inner circle is associated with the z-score (blue, downregulated; red, upregulated).

The GO enrichment analysis of the differentially expressed UniGenes displayed interesting data, which are shown in a Manhattan plot (Fig. 4A; Table S7). In the biological processes category, 83 GO terms were identified as enriched functional categories, and we selected eight primary or highly representative categories. We suggest that these categories not only effectively explain the biotic processes related to the global changes in transcriptomic events but also summarize some redundant terms (at different GO levels). The eight categories were as follows: response to stress (GO:0006950, 536 UniGenes), response to hormones (GO:0006950; 263 UniGenes), oxidation-reduction process (GO:0055114; 275 UniGenes), organization and biogenesis of cell wall processes (GO:0071554; 104 UniGenes), response to abscisic acid (GO:0009737; 100 UniGenes), polysaccharide metabolic process (GO:0005976; 82 UniGenes), response to water deprivation (GO:0009414; 68 UniGenes), and photosynthesis (GO:0015979; 47 UniGenes). The UniGenes contained in each of these categories are listed in Table S8. In addition to these eight categories, Table S7 shows all GO terms enriched in biological processes, and the five categories with the highest number of differentially expressed UniGenes can be identified. Those five categories were the following: cellular process (GO:0009987; 1,371 UniGenes), metabolic process (GO:0008152; 1,133 UniGenes), organic substance metabolic process (GO:0071704; 1,160 UniGenes), cellular metabolic process (GO:0044237; 1,133 UniGenes), and primary metabolic process (GO:0044238; 1,061 UniGenes). Additionally, enriched KEGG pathways were identified for the avocado UniGenes (Table S9). These data revealed that biosynthesis of secondary metabolites (KEGG:01110; 1,008 UniGenes), phenylpropanoid biosynthesis (KEGG:00940; 279 UniGenes), flavonoid biosynthesis (KEGG:00941; 144 UniGenes), phenylalanine metabolism (KEGG:00360; 72 UniGenes), and photosynthesis (KEGG:00195; 26 UniGenes) were the most significantly enriched pathways (Table S9).

Moreover, in Figs. 4B–4E, the circle plots represent the genes included in the eight enriched categories that were selected as relevant in the avocado stem-*F. kuroshium* infection process. The radar chart shows the distribution of individual GO terms at 1, 4, 7 and 14 dpi, and no significant differences between the number of genes that were up- and down-regulated were observed. The GO terms that changed the most over all the infection stages were response to stress (GO:0006950), oxidation-reduction process (GO:0055114), and response to hormones (GO:0009725); 226, 112 and 101 UniGenes were upregulated and 198, 117 and 109 UniGenes were downregulated for these GO terms, respectively.

The UniGenes included in the eight selected enriched GO terms were also identified in the different clusters. The data showed 1,475 UniGenes in total, and we detected that 235 belonged to cluster 1, 404 to cluster 2, 138 to cluster 3, 328 to cluster 4, 188 to cluster 5 and 182 to cluster 6. To analyze the infection process in more detail, we surveyed the 12 UniGenes that showed the greatest change in expression level (6 UniGenes that were upregulated and 6 UniGenes that were downregulated) in each of the six distinct clusters of differentially expressed UniGenes with different expression patterns (Fig. S3). The UniGenes selected are listed in Table S10, and their relevance is described in the discussion section.

### Avocado immune PRRs and NLRs

To contribute to knowledge related to immune receptors that may participate in the avocado defense response against *F. kuroshium*, we identified in the avocado data two types of receptors: the pattern recognition receptors (PRRs) and nucleotide-binding domain and leucine-rich repeat-containing proteins (NLRs), which active PAMP-triggered immunity (PTI) and effector-triggered immunity (ETI), respectively.

Regarding PRRs, either by the annotation of their corresponding homologs or based on the presence of some characteristic motifs, a total of 223 UniGenes were identified as transcripts codifying for PRRs (Table S11). Interestingly, only 10 of these UniGenes (4.48%) were differentially expressed (Table S12). The UniGenes UN009421, UN019209, UN021431 and UN025891 belong to cluster 2. UN009421 is homolog to CoAc2 (AT4G26180) and encodes a mitochondrial CoA transporter. UN025891 encodes a plasma membrane-localized leucine-rich repeat receptor kinase involved in brassinosteroid signal transduction. UN019209 and UN021431 UniGenes are homologs to a LysM motif-containing F-box protein (AT1G55000). The six remaining differentially expressed UniGenes showed distinct expression patterns and encoding to homologs of RKL1, namely, AT1G48480 (UN004766); PR5K; AT5G38280 (UN010960); and AT2G19130 (UN004495, UN020702, UN021172, and UN061464), which is a member of a multigene family that encode to no well characterized G-type lectin S-receptor-like serine/threonine-protein kinase.

**Figure 5:**
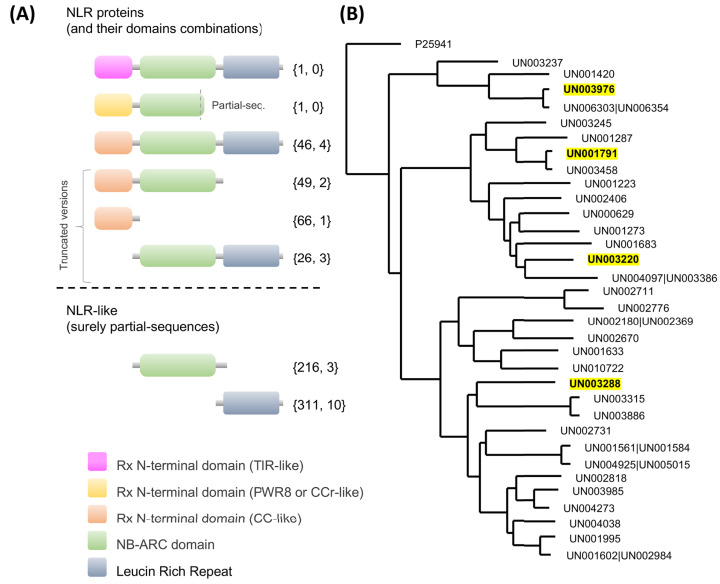
Avocado nucleotide-binding leucine-rich receptors (NLRs). (A) Primary structures of the NLR proteins in which their characteristic conserved domains in a stereotypical configuration are shown in colored boxes (a variable N-terminal domain (TIR, CCr or CC)), a central nucleotide-binding site (NBS) domain similar to the AAA-ATPase family and a C-terminal leucine-rich repeat (LRR) domain; more details in Fig. S4. Truncated versions and partial sequences are also shown. The total number of UniGenes classified as a specific NLR type and the number of those identified as differentially expressed are shown in curly brackets. (B) Maximum likelihood tree depicting the phylogenetic relationships of avocado UniGenes classified as NLR proteins. All but one of the protein sequences were removed from the tree if they showed an identity equal to or greater than 90% with other NLR proteins and if, in a previously created phylogenetic tree, they were grouped in a monophyletic clade (in the phylogeny, those identifiers are shown after a pipe or vertical bar). Highlighted identifiers (bold letters and yellow color) represent differentially expressed UniGenes.

Regarding NLRs, key players in plant defense which recognize pathogen effectors and trigger immunity (Van Wersch et al., 2020), a total of 716 UniGenes contained at least one of the NLR characteristic domains in their coding region (Fig. 5A; Table S13). Interestingly, one unique UniGene (UN000626) encoded a TIR-type NLR; this protein seems to be complete since the conserved domains were identified in the structure. Another unique UniGene (UN036979) was identified as a putative PWR8-type NLR; in this case, the protein sequence seems to be partial, and only the N-terminal domain PWR8-type (CCr) and the central NBS domain were identified. The rest of the sequences were classified as follows: full CC-NLR proteins, 46 UniGenes; CC-NBS- and CC- truncated versions, 44 and 66 UniGenes, respectively; NLR proteins in which none of the canonical variable N-terminal domains (TIR, CC or CCr) could be identified, 26 UniGenes; and partial sequences in which the translated coding region contained only an NBS domain or an LRR domain, 226 and 311 UniGenes, respectively (Fig. 5A; Table S13).

Considering that mainly CC-type NLR receptors were identified in our avocado transcriptome, we resolved the phylogenetic relationships within these families (Fig. 5B). Only UniGenes that encode full CC-NLR proteins were considered. In addition, specific functional motifs were also identified, such as the MADA motif, which is evolutionarily conserved and is present in a certain percentage (~20%) of the total CC-NLR protein-coding genes in plants. Interestingly, in avocado, the MADA motif was clearly identified in a high percentage of the full CC-NLR proteins (67.39%, 31 proteins; Fig. S4). In the remaining proteins, the MADA motif was absent or it was unclear whether it was present because short sequences were missing in the N-terminal domain and near the start codon.

We also identified the EDVID motif or the EDVID-like motif (Fig. S4). Based on the results presented here, avocado CC-NLR proteins, like those in other plant species (Lukasik & Takken, 2009; Meyers et al., 2003; Wróblewski et al., 2018), can be classified into at least three major groups based on this EDVID-like motif (Fig. S4).

Regarding the NBS domains, we observed conserved motifs (p-loop, kinase, RNBS, GLPL, MHD; Fig. S5) that are crucial for ADP/ATP binding and shifting (Danot et al., 2009; Van Ghelder et al., 2019) and are involved in intra- and extramolecular interactions (Moffett et al., 2002; Van Ooijen et al., 2008). It has been consistently proven that, despite some variations, these motifs are conserved in all NLR subfamilies (including those present in gymnosperms) and in the proteins of prokaryotes with an NBS domain (NB-ARC), which do not display upstream N-terminal domains (Yue et al., 2012). Interestingly, only four UniGenes that encoded full CC-NLR proteins were identified as differentially expressed in response to *F. kuroshium* infection (Fig. 5B; Tables S8 and S13). Based on their expression profiles (Fig. S3), two of them, UN003976 and UN001791, belong to cluster 3, which contains differentially expressed UniGenes with maximum upregulation at 7 dpi. UN003288 belongs to cluster 4, a group of genes whose expression pattern increased over time, and UN003220 belongs to cluster 1, corresponding to UniGenes that show a rapid increase in expression level at 1 dpi and then a gradual decrease in expression.

**Figure 6:**
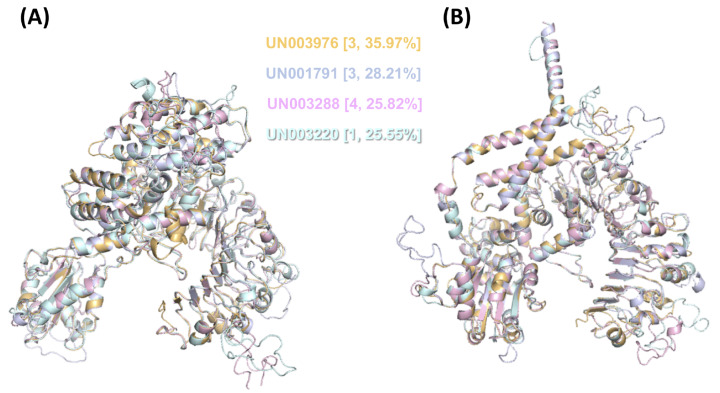
3D structural model superposition of the four CC-NLR proteins that respond to *F. kuroshium* infection in avocado. UN003976 (light orange), UN001791 (light blue), UN003288 (light pink), and UN003220 (pale cyan). Their inactive (A) and active (B) forms were modeled based on the available structure of the *A. thaliana* ZAR1 protein, which has been reconstituted as part of the ZAR1-RKS1-PBL21 resistosome (Wang et al., 2019a) and is available in the Protein Data Base (PDB entries 6j5t.1.A.pdb and 6j5t.1.C.pdb, respectively). In (A) and (B), the cluster number to which each UniGene belongs based on its expression profile (Fig. S3) and the percentage of identity that it shows with respect to *A. thaliana* ZAR1 are shown in curly brackets.

Then, we performed modeling of both their inactive and active forms based on the *A. thaliana* orthologue ZAR1 (Fig. 6). We noticed that the CC-type N-terminal domain was complete only in UN001791 and UN003288, and the MADA motif was clearly distinguishable in these UniGenes (Fig. S4). These two CC-NLR proteins were also independently modeled in a structure consisting of five monomers (Fig. S6), mimicking the core region of the ZAR1-RKS1-PBL21 reconstituted resistosome (Wang et al., 2019a).

Interestingly, an avocado orthologue to *A. thaliana* RKS1 protein was not identified in any of the OrthoMCL-defined protein families (Table S5). RKS1 is a member of an OrthoMCL-defined protein family (OrthoGroup02788) containing a total of 22 proteins (7 proteins from *A. thaliana* (including RKS1), 5 from *P. persica*, 7 from *V. vinifera*, and 3 from *S. lycopersicum*). Notably, orthologues were absent not only in avocado but also in monocots (*Z. mays* and *M. acuminata*) and *A. trichopoda*, a member of the most ancient known clade of flowering plants. The PBL21 protein is a member of OrthoGroup02770, another family that also contains a total of 22 orthologous proteins but is apparently present in all reference plants used in this study (4 from *A. thaliana*, 2 from *P. persica*, 1 from *V. vinifera*, 3 from *S. lycopersicum*, 2 from *Z. mays*, 6 from *Musa acuminata*, 1 from *A. trichopoda*, and 3 from avocado (*P. americana*)). Despite its presence in the avocado transcriptome, none of the orthologue UniGenes were identified as differentially expressed in response to *F. kuroshium* infection.

### Identification of fungal transcripts present in avocado stems

**Figure 7:**
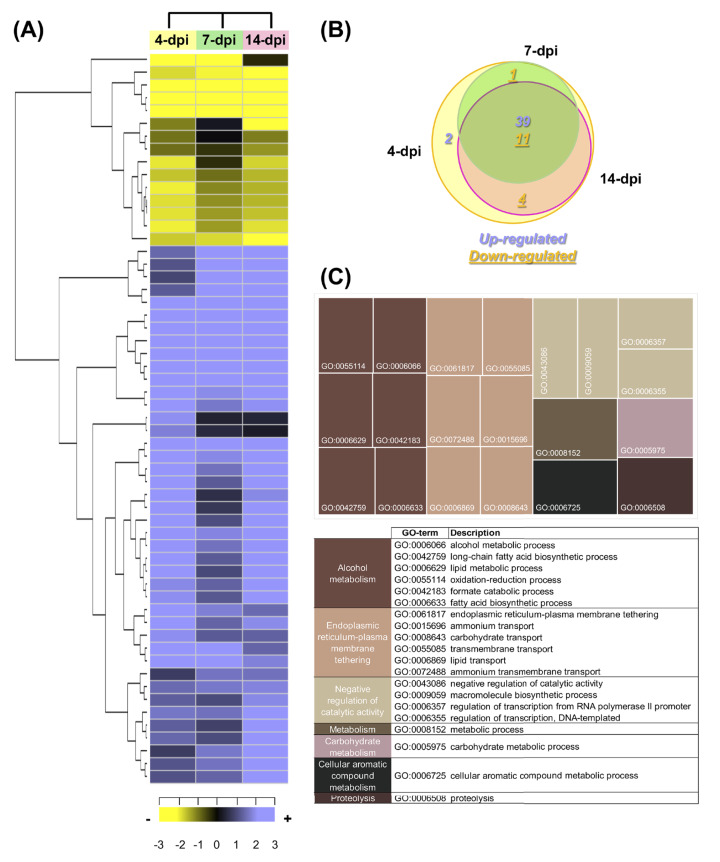
Expression profiling of the differentially expressed *F. kuroshium* genes. (A) Heat map showing the hierarchical clustering analysis of differentially expressed genes that were identified after comparing 4, 7, and 14 dpi vs 1 dpi. Pearson correlation distance metrics and average linkage clustering methods were used. As shown on the color scale at the bottom of the figure, yellow indicates downregulation (−), purple indicates upregulation (+), and black indicates unchanged expression. The Venn diagrams (B) show common or unique UniGenes that were identified as differentially expressed at the sampled time points. The number of genes up- or downregulated at each time point is shown using purple or yellow numbers, respectively. (C) REVIGO treemap (Supek et al., 2011) summarizing the overrepresented Gene Ontology biological process categories in the list of differentially expressed *F. kuroshium* genes. The size of each rectangle is proportional to the number of annotations in that category.

According to the criteria (a log_2_FC value ± 1.0 and an adjusted *p*-value of ≤ 0.05), a total of 57 *F. kuroshium* genes were identified as differentially expressed during the plant-fungi interaction and the development of infection (Table S14). Heat map and Venn diagram analyses at 4, 7 and 14 dpi showed that more than 50% of genes maintained a similar expression profile over time; moreover, 74% of the genes were upregulated and 26% were downregulated (Fig. 7A and 7B, respectively). Orthologues (and paralogs) of these *F. kuroshium* genes were identified in the genomes of *Nectria haematococca* (commonly referred to by its asexual name, *F. solani*), *F. fujikuroi*, *F. oxysporum*, *F. verticillioides*, *F. pseudograminearum* and *F. graminearum*. Interestingly, the copy numbers of these genes were variable. According to the annotation, fourteen differentially expressed *F. kuroshium* genes had no functional or descriptive assignment (uncharacterized proteins). For the rest of the genes, the enriched GO terms within the biological process category were summarized using the REVIGO tool (Supek et al., 2011). The summary showed 13 genes for “alcohol metabolism” and 15 genes for “endoplasmic reticulum-plasma membrane tethering” (Fig. 7C). Additionally, “negative regulation of catalytic activity”, “metabolism”, “carbohydrate metabolism”, “cellular aromatic compound metabolism” and “proteolysis” were represented by fewer than 4 genes each (Fig. 7C). To identify genes related to pathogenicity, each protein was compared by the BLASTp algorithm against the pathogen-host interaction database. The analysis showed that 21 expressed *F. kuroshium* genes (37%) had significant similarity (*e*-value ≥ 10-6) to some PHI-base sequences, and the mutant phenotype observed in other pathogenic fungi showed reduced virulence, suggesting that these genes play an important role during pathogenesis (Table S14).

The comparison of the three data sets (4, 7 and 14 dpi) in the Venn diagram indicated that 39 up- and 10 downregulated genes were shared among the three stages of infection, 4 downregulated genes were shared between 4 and 14 dpi, 1 upregulated gene was shared between 4 and 7 dpi and one gene was only upregulated at 4 dpi. The only two genes upregulated at 4 dpi were Scf14_Gene-0.24 and Scf41_Gene-21.18, which were annotated as “hypothetical protein like phosphatidyl synthase” and “aldehyde dehydrogenase”, respectively. Scf27_Gene-18.41 was downregulated at both 4 and 7 dpi and was annotated as “probable endoglucanase type K”; this gene showed the greatest decrease in expression on both days. Scf201_Gene-0.1, Scf38_Gene-0.53, Scf71_Gene-7.29 and Scf135_Gene-0.11 were downregulated at 4 and 14 dpi and were identified as “uncharacterized protein”, “ammonium transporter MEPa”, “CAP20-virulence factor” and “urea transporter DUR3”, respectively. Among the genes upregulated at all three stages of infection, Scf30_Gene-0.42 exhibited the greatest change in expression and was annotated as “alcohol oxidase”, and Scf38_Gene-1.54 and Scf9_Gene-3.23 displayed the highest expression at 4 dpi and were both annotated as uncharacterized proteins. Scf15_Gene-17.40, Scf63_Gene-21.71 and Scf25_Gene-1.60 were annotated as “hypothetical protein similar to extracellular invertase”, “serine-type carboxypeptidase F precursor” and “related to tetracycline resistance proteins”, respectively, and displayed the highest expression at 7 dpi. Finally, at 14 dpi, Scf2_Gene-8.62, “related to allantoate permease”; Scf61_Gene-6.97, “related to nonribosomal peptide synthetase”; and Scf14_Gene-8.44, “uncharacterized protein” exhibited the highest expression. The genes that were the most downregulated at all infection stages were Scf15_Gene-2.40, annotated as “hypothetical uncharacterized protein”; Scf7_Gene-2.51, annotated as “related to glycosyl hydrolase family protein”; and Scf13_Gene-3.51, annotated as “related to uracil permease”.

## Discussion

### The avocado- *Fusarium kuroshium* pathosystem

FD is a disease that is independently caused by two distinct complexes formed by ambrosia beetles that are morphologically related to *Euwallacea fornicatus* (Eichhoff) (Coleoptera: Curculionidae: Scolytinae), the polyphagous shot hole borer (PSHB) and KSHB, and their respective fungal symbionts (Carrillo et al., 2016; Eskalen et al., 2012, 2013; Mendel et al., 2012; Na et al., 2017). The complexes have a broad host range, including landscape and agricultural trees, particularly avocado trees (Carrillo et al., 2016; Eskalen et al., 2013; Na et al., 2017). The relevance of FD has led to the identification of the fungal symbionts associated with each insect; for PSHB, the three pathogenic fungal symbionts are *F. euwallaceae*, *G. euwallaceae* and *Paracremonium pembeum* (Freeman et al., 2013; Lynch et al., 2016), and for KSHB, the associated fungi are *F. kuroshium* and *G. kuroshium*.

Here, we developed a reproducible, practical and low-cost model system consisting of Hass avocado stems inoculated with *F. kuroshium*. This system allowed us to control some biotic and abiotic variables to have confidence in the molecular data, which was the main goal of this research. *F. kuroshium* pathogenicity under in vitro or greenhouse conditions has been analyzed only by Na et al. (2017). They tested the pathogenicity of the fungus in 2-year-old avocado cv. Zutano and reported the mean lesion length of xylem discoloration; there was a clear difference from the control but less pathogenicity than was observed with *F. euwallaceae*. For our pathosystem, we selected the Hass avocado variety since it is the most important cultivated and marketed avocado variety in Mexico. We injured avocado stems to mimic the ambrosia beetle and to inoculate the fungus directly into the vascular tissue. Similar to a previous report (Na et al., 2017), we detected that the avocado stems displayed brownish discoloration starting in the early infection stages (1 and 4 dpi), and the discoloration increased over time (Fig. 1). In contrast to the data obtained by the Eskalen research group in whole trees, we detected a white exudate exclusively in the infected avocado stems at 7 and 14 dpi (Fig. 1). The white exudate has been recognized as a typical symptom of FD in some but not all host trees. This phenomenon may be associated with vessel occlusion, resulting in the formation of tyloses or with the deposition of gums composed of phenols, pectin, and lipids that are already recognized as a response of some trees to various stimuli, including mechanical damage and vascular pathogen infection (Inch et al., 2012; Veronica De et al., 2016). It will be interesting to characterize the precise chemical nature and properties of this white exudate associated with the response of Hass avocado stems to *F. kuroshium* infection in future studies and to analyze the implications of the anatomical changes during the production of this exudate. Additionally, it would be relevant to coinfect avocado stems with *G. kuroshium* and *F. kuroshium* to understand whether this coexistence potentiates the pathogenicity capabilities of both fungal pathogens.

### Major gene categories involved in avocado defense responses to infection by *F. kuroshium*

The avocado transcriptomics data were generated mainly to investigate molecular changes related to the avocado defense response after inoculation with the *F. kuroshium*. Both groups of differentially expressed UniGenes (up- and downregulated) showed, in general, oscillatory behavior (Fig. 3). Overall, our analysis indicated that plant defenses were activated and that the defense response persisted in the avocado stems at later stages but was not sufficient to prevent infection from progressing. These increases and decreases in differential UniGene expression may fluctuate, similar to the changes in their expression profiles over time (Fig. 3; Fig. S3). Moreover, the fluctuations were also evident by the six clusters generated.

Our analysis was focused on the UniGenes within the different clusters that were well identified based on the *A. thaliana* annotation and showed their major expression changes. The UniGenes that displayed upregulation are as follows. In Cluster 1, an ABC transporter F family member 1-like protein was identified that belongs to one of the largest protein families and is implicated in various processes, such as pathogen response and phytohormone transport (Kang et al., 2011). Information regarding this type of transporter in bacteria related to ABC-F transporter function indicated that they play an important role in antibiotic resistance and ribosome protection (Ero et al., 2019); this result combined with our data suggests that in avocado, they play an important role in ribosome protection and in toxic compound resistance during infection with *F. kuroshium*. Another family of proteins that was likely involved in avocado infection was the peptidyl-prolyl cis-trans isomerase family (PPiase). These proteins are involved in protein folding processes and are recognized in at least three families, namely, cyclophilins, FK506-binding proteins (FKBPs) and parvulins (He, Li & Luan, 2004). In the *A. thaliana* genome, there are 29 PPiases (Romano, Horton & Gray, 2004), and their expression is induced by fungal infection (Godoy et al., 2000) and hormones (Romano, Horton & Gray, 2004). Furthermore, *A. thaliana* knock-out mutations in three immunophilins (AtCYP19, AtCYP57, and one FKBP) result in an increased susceptibility to *P. syringae* (Pogorelko et al., 2014). Similar results were observed when cotton cyclophilin was overexpressed in tobacco, which conferred higher tolerance to both salt stress and *P. syringae* infection (Zhu et al., 2011).

In cluster 2, UN028398 (not identified) was the most highly upregulated, followed by UN023639, which encodes the mediator complex subunit Med28. The mediator is a protein complex that functions as a moderator between transcription factors and transcription activation by RNA polymerase II (Björklund & Gustafsson, 2005; Kornberg, 2005). Mediator studies in *A. thaliana* plant Med12, Med25 and Med8 mutants showed that the mutation enhanced susceptibility to *Botrytis cinerea* and *A. brassicicola*. Additionally, the Med16 mutant was defective in cold acclimation to freezing temperatures and was compromised in salicylic acid-induced PR gene expression as well as resistance to *P. syringae* (Wathugala et al., 2012).

Moreover, Med16 plants had reduced PR-1 levels and exhibited impaired systemic acquired resistance (Fu & Dong, 2013). In cluster 3, UniGene UN003714, encoding glutamine-dependent asparagine synthase 1 (ASN1), was the most upregulated. ASN1 is involved in the synthesis of L-asparagine from glutamine and has an important function in nitrogen metabolism and defense responses to microbial pathogens; in transgenic *A. thaliana* plants, overexpressing pepper asparagine synthetase resulted in enhanced resistance to *P. syringae*. Moreover, ASN1 induction can have opposite effects against the hemibiotrophic bacterial pathogen *Xanthomonas campestris* depending on the time post-infection (Hwang, An & Hwang, 2011). Finally, it has been proposed that ASN1 is involved in metabolic changes that facilitate host cell death during plant-pathogen interactions (Seifi et al., 2014). In cluster 4, the UniGene encoding multidrug resistance-associated protein 10 was the most upregulated. Multidrug resistance-associated proteins (MRPs) are a subfamily of full-molecule ABC transporters (Rea, 2007) that have an integral role in the plant detoxification mechanism (Klein, Burla & Martinoia, 2006). They work via the sequestration vacuole (“excretion storage”) to remove both endogenous and xenobiotic compounds such as glutathione conjugates (Klein, Burla & Martinoia, 2006; Rea, 2007).

Moreover, two maize MRPs are important in the transport of the anthocyanin pigment into the vacuole (Goodman, Casati & Walbot, 2004). Additionally, MRPs can transport other amphipathic anions, including glucuronate conjugates, linearized tetrapyrrole catabolites, folate and its derivatives (Rea, 2007). This evidence suggests the important role of MRPs in the plant-fungus interaction. The UniGene in cluster 5 with the highest expression level encodes a pentatricopeptide repeat (PPR) superfamily protein and has been recognized for its role in plant growth and development (Xing et al., 2018). Additionally, significant functions for several PPRs in response to biotic and abiotic stresses have been identified, such as functions in photooxidative stress responses (Karpinski et al., 1999) and ROS stress responses (Zsigmond et al., 2008) as well as in isoprenoid biosynthesis. Finally, the most highly upregulated UniGene in cluster 6 encodes a kinesin-13A (a microtubule-based motor protein) that participates in regulating the branching pattern of leaf trichomes (Li et al., 2017; Lu et al., 2005) in *A. thaliana* and *Gossypium hirsutum*. Trichomes serve as physical barriers against insect attack and fungal infection (Levin, 1973). Taken together, these data on the upregulated UniGenes suggest that in avocado stems during *F. kuroshium* infection, part of the defense response is related to both detoxification and protection mechanisms.

In addition, we analyzed the genes that displayed the greatest downregulation in each of the six clusters identified. In cluster 1, a nucleolar protein, gar2 (UN023639), named NSR1 in yeast and exhibiting high similarity to parallel1 (Par1; (Petricka & Nelson, 2007)), was found; in *A. thaliana*, parl1 mutants exhibit auxin-dependent growth defects and aberrant leaf venation. No direct information regarding its function during interactions with fungal pathogens has been obtained. In cluster 2, UN083934 and UN004830 encoded ubiquitin 11. Ubiquitin 11 is part of the ubiquitin–proteasome system, and its important function in plant-pathogen interactions is well documented (Miricescu, Goslin & Graciet, 2018). UN007206 was the most downregulated in cluster 3 and encodes light-regulated zinc finger protein 1, a transcriptional factor called LZF1, STH3, DBB3 and BBX22 (Khanna et al., 2009), and it modulates early chloroplast development and anthocyanin accumulation (Chang et al., 2008). These observations suggest that BBX22 could participate in the response to pathogens, since anthocyanins play an important role in protecting plant cells against damage by reactive oxygen species and are the first sign of programmed cell death (Dauphinee et al., 2017). In cluster 4, the UniGene for PHE ammonia lyase 1 (PAL) was the most downregulated. PAL catalyzes the first step in the phenylpropanoid pathway and is involved in the synthesis of many important metabolites, flavonoids, phenylpropanoids, lignin, and glyceollins (soybean-derived phytoalexins; Zhang et al., 2017). Many studies have demonstrated the important function of PAL during the defense response in different pathosystems (Abbasi, Graham & Graham, 2001; Cass et al., 2015; Kim & Hwang, 2014). In cluster 5, the most downregulated UniGene encodes a member of the amino acid permeases (AAPs) within the subfamily of amino acid/auxin permeases. AAPs are tissue-specific proteins that predominantly transport neutral and acidic amino acids with moderate affinity (Wan et al., 2017). No direct evidence relates AAPs to fungal biotic stress. However, it is obviously involved in important events related to the nitrogen mobilization pathway, in which amino acid transporters are associated with plant responses to pathogens (Sonawala et al., 2018). However, a protein with unknown function was the most downregulated in cluster 6, followed by MLP-like protein 423, a member of the major latex protein family; these proteins belong to the Bet v1 family, also known as the pathogenesis-related 10 (PR10)-like protein family (Wang et al., 2015). Recently, He and collaborators described the biological role of MLP-like protein 423, finding that its expression was inhibited by *Botryosphaeria berengeriana* f. sp. *piricola* and *A. alternata* apple pathotype infection (He et al., 2020). Additionally, the overexpression of MLP-like protein 423 in apple calli resulted in lower expression of resistance-related genes, and RNA-seq analysis indicated that MLP-like protein 423 negatively regulates apple resistance to infections by inhibiting the expression of defense and stress-related genes, as well as transcription factors. The identity of each of the UniGenes identified as downregulated suggests that the number of certain proteins is modulated through general regulation pathways, such as proteasome and transcription pathways. Finally, the combined data for the up- and downregulated UniGenes showed changes in avocado nitrogen metabolism.

### Avocado immune receptors expression during *F. kuroshium* interactions and in the resistosome

Recognition of pathogen invasion through immune receptors is performed mainly by two structurally different proteins located on different subcellular compartments. One is the plasma membrane-localized PRRs that perceive extracellular pathogen-associated molecular patterns (PAMPs) activating PAMP-triggered immunity (PTI) (Boutrot & Zipfel, 2017). The other type of proteins is composed of intracellular receptors of the nucleotide-binding domain leucine-rich repeat protein superfamily (NLRs) (Jones, Vance & Dangl, 2016), which induces the effector-triggered immunity (ETI), which is often accompanied by hypersensitive response (HR) cell death. Together, these immune receptors act as a network of surveillance machines that recognize extracellular and intracellular pathogen invasion-derived molecules, ranging from conserved structural epitopes to virulence-promoting effectors (Kim & Castroverde, 2020).

Membrane-bound plant PRRs include receptor-like kinases (RLKs) (Shiu & Bleecker, 2003) that have an extracellular domain, such as leucine rich repeats (LRRs), lectin, lysine motif (LysM) or wall-associated kinases (WAK), with a single transmembrane spanning region and a cytoplasmic kinase domain;receptor- like proteins (RLPs) (Wang et al., 2008), which possess an extracellular LRR domain and a C-terminal membrane anchor but lack the cytoplasmic kinase domain; and polygalacturanase-inhibiting proteins (PGIP) (Di Matteo et al., 2003), which have only an extracellular LRR domain.

During *F. kuroshium* interaction in avocado, a total of 223 UniGenes were identified as PRRs, but only ten were selected as differentially expressed. Interestingly, UN025891 encodes a plasma membrane-localized leucine-rich repeat receptor kinase involved in brassinosteroid signal transduction (ATBRI1; AT4G39400). Additionally, it has been proven that disease resistance caused by some bacterial and fungus species is augmented in barley lines modified in the brassinosteroid receptor BRI1 (Ali et al., 2014). UN019209 and UN021431 UniGenes are homologs to a LysM motif-containing F-box protein (AT1G55000). These proteins (with F-box+LysM combination) are found not only in angiosperm plants but also in mosses and in green algae (Zhang, Cannon & Stacey, 2009). With a few exceptions, these proteins are single-copy genes and highly conserved in the plant kingdom, which implicates a conservation of their biological function, which is still unknown. Due to the presence of the LysM domain, it has been speculated that these proteins can recognize glycoproteins and presumably participate in their degradation (Zhang, Cannon & Stacey, 2009). Moreover, recently, in *Lotus japonicas*, receptors associated with nodulation factors and chitin were found to have a very similar structure but contain two diverging motifs in the LysM1 domain that are necessary for discriminating between immunity and symbiotic functions (Bozsoki et al., 2020).

The six remaining differentially expressed UniGenes associated with PRRs show distinct expression patterns and encoding to homologs of RKL1 (UN004766), PR5K (UN010960) and AT2G19130 (*A. thaliana* G-type lectin receptor kinases; UN004495, UN020702, UN021172, and UN061464). RKL1 is a salicylic acid-responsive gene that has been suggested to mediate defense responses (Ohtake, Takahashi & Komeda, 2000). In *A. thaliana*, the PR5K is a polypeptide with the structural and biochemical features of a transmembrane receptor protein that is possibly involved in the perception of microbial signals (including fungus) (Wang et al., 1996). Finally, the G-type lectin S-receptor-like serine/threonine-protein kinase (G-LecRKs) includes 38 members that are represented in *A. thaliana* (Teixeira et al., 2018) and contain an α-mannose binding bulb lectin domain. LORE is a member of G-LecRKs that can directly recognize bacterial lipopolysaccharide (Ranf et al., 2015). Therefore, the participation of the G-LecRks in the avocado defense processes may be relevant.

With respect to NLRs, four UniGenes (UN003976, UN001791, UN003288, and UN003220) encoding CC-type NLR proteins were identified as differentially expressed in response to *F. kuroshium* infection (Figs. 5B and 6; Table S13). Interestingly, these proteins are transcriptionally active mainly at early (1 dpi) and later (7 and 14 dpi) stages of infection. This activity pattern reflects their role during pathogen recognition, defense response activation and disease establishment, which provoked symptoms such as discoloration and vascular tissue damage (Fig. 1). These results are consistent with those of previous reports showing that genes encoding NLR proteins show distinct expression profiles and even tissue-specific expression patterns (Adachi, Derevnina & Kamoun, 2019); therefore, it is argued that plants have evolved tissue-specific NLR networks in a species-dependent manner, possibly to match specific pathogen attacks on different organs and tissues (Deng et al., 2017).

At least two of these four CC-type NLR proteins identified in avocado that were differentially expressed in response to *F. kuroshium* infection were modeled (Fig. S6) based on the structure of the ZAR1-RKS1-PBL21 *A. thaliana* resistosome (Wang et al., 2019a). The ZAR1-RKS1-PBL2 resistosome structure reveals how NLR immune receptors are activated. The active intermediate state of ZAR1 (Wang et al., 2019b) forms a wheel-like pentamer and resides in a 1:1 heterodimeric complex with RKS1. When a PBL2 protein (modified by the AvrAC effector of *Xanthomonas campestris*) is added to the ZAR1:RKS1 complex, it can be found in a different PBL2-induced conformation (Wang et al., 2019a).

Interestingly, these four avocado NLR receptors, which are orthologues to ZAR1, can be properly modeled with their active and inactive forms (Fig. 6) and even form a wheel-like pentamer (Fig. S6); no orthologues from RKS1 were detected in the avocado transcriptome. The results suggest that in avocado, as in other plant species (e.g., monocots (*Z. mays* and *M. acuminata*) and *A. trichopoda*), orthologues to RKS1 are absent. These observations are consistent with previous reports showing that ZAR1 can indirectly recognize other unrelated bacterial effector proteins (e.g., PBL2 modified by the AvrAC effector of *X. campestris*), all through an association with closely related pseudokinases that belong to receptor-like cytoplasmic kinase subfamily XII-2 (RKS1, ZED1 and ZRK3), respectively (Lewis et al., 2013; Seto et al., 2017; Wang et al., 2019a). Together, these results suggest that in avocado, even when some CC-type NLR proteins form a similar structure to the ZAR1-RKS1-PBL2 resistosome and even perform a similar function, specific proteins may be required to form a complex that represents the structure of the resistosome in its active intermediate state. Regarding avocado orthologues to PBL2, some of them (only partial sequences) can be identified in the avocado transcriptome; however, all of them were differentially expressed. This is expected considering that PBL2 needs to be modified by AvrAC effector recognition; until now, no plant serine/threonine-protein kinase (similar to PBL2) structure has been resolved in the presence of fungal effectors.

Regarding the domains and motifs present in CC-type NLR proteins, not only the highly conserved domains but also the motifs present on each of the domains play important roles in the structural conformation of resistosomes, and these motifs and domains are required for resistosome function. For example, RKS1 interacts with the ZAR1 LRR domain; it has been predicted that the LRR domain is the autoinhibitory region in the inactive state and is the sensor during the activation process (Burdett et al., 2019). The LRR domain was identified in all UniGenes encoding full CC-NLR proteins (Fig. 5A). Regarding the NBS domain, although the only known 3D structure other than ZAR1 is from the tomato protein NRC1 (Steele, Hughes & Banfield, 2019), we demonstrate that at least the differentially expressed avocado UniGenes that encode CC-type NLR proteins can be modeled in both their active and inactive forms (Fig. 6) when the resolved 3D structures for ZAR1 (PDB: 6j5t.1. A and 6j5t.1. C) are used as templates. In addition, the superposition of the modeled structure of these proteins shows that, as in the available structures from NRC1 and ZAR1, the P-loop (kinase a), kinase 2, and RNBS-A, -B, -C and -D motifs adopt analogous positions, and the orientation of the ADP nucleotide is virtually identical (Fig. 6) (Wang et al., 2019a). The similarities in the structures also extend to other conserved regions, such as GLPL and MHD motifs (Fig. S5; Fig. 6) (Van Ghelder et al., 2019).

Regarding the CC-type N-terminal domain, we note the presence of the EDVID motif (or the EDVID-like motif corresponding area) in all full CC-NLR proteins (Fig. S4). This motif has been shown to be important for intramolecular interactions between the NBS and LRR domains (Bentham et al., 2018), and it is necessary for both the autoinhibition and activation of the protein (Burdett et al., 2019). Finally, after confirming the presence of the MADA motif in a high percentage of the full CC-NLR proteins (Fig. S4), including the differentially expressed UN003976 and UN001791 (Fig. S6), we suggest that at least these two CC-type NLR proteins in avocado are involved in the defense mechanism induced against *F. kuroshium* infection.

### Candidate genes involved in *F. kuroshium* pathogenesis

*F. kuroshium* is vectored by the ambrosia beetle and gains access to the plant to colonize the host xylem. According to Hulcr & Stelinski (2017), FD is the consequence of mass *F. kuroshium* accumulation on stressed trees. However, it remains unknown whether *F. kuroshium* contributes to disease development through an active pathogenic mechanism. In answering this question, it is important to consider that *F. kuroshium* belongs to a widely recognized genus of necrotrophic phytopathogens that can produce effectors, toxins and hydrolytic enzymes that, in combination, provoke host cell death (Ma et al., 2013). The notion of *F. kuroshium* as an active pathogen is supported by the fact that *F. kuroshium* infects healthy avocado tissue (Na et al., 2017) and was reaffirmed by the present study, in which the transcriptome coverage allowed us to identify 57 genes that are differentially expressed in *F. kuroshium* during the infection process. Interestingly, the analysis revealed that alcohol metabolism is strongly involved in the *F. kuroshium* infection process in avocado stems, as the most upregulated genes were associated with this GO term (Fig. 7). The enzymes encoded by these genes are alcohol oxidases, 5-diketo-D-gluconic acid reductase, aldehyde dehydrogenase, protocatechuate 3,4-dioxygenase beta subunit, choline dehydrogenase, and cellobiose dehydrogenase. There are few reports of these enzymes in filamentous fungi, which is additional evidence regarding their roles during pathogenesis. For example, alcohol oxidases are flavoenzymes that catalyze the oxidation of alcohols to carbonyl compounds, producing hydrogen peroxide (H_2_O_2_) (Couderc & Baratti, 1980; Cregg et al., 1989; Koch et al., 2016). However, the lack of an alcohol oxidase negatively impacts *Cladosporium fulvum* pathogenicity in tomato plants (*Lycopersicon esculentum* L.) (Segers et al., 2001). The role of this type of enzyme is not yet clear, and we hypothesize that it detoxifies the ethanol content that is produced in avocado tissue during infection and that H_2_O_2_ release may impact the redox environment. In our study, Scf30_Gene-042, which encodes an alcohol oxidase, was strongly upregulated during all three infection stages. Another interesting enzyme was aldehyde dehydrogenase (Scf41_Gene-21.18), which catalyzes the irreversible oxidation of endogenous and exogenous aldehydes to nontoxic carboxylic acids. In *Magnaporthe grisea*, two putative family-four aldehyde dehydrogenase genes were silenced by RNAi, severely compromising the pathogenesis of the rice blast fungus. Additionally, the mutant strains were highly sensitive to membrane stress (Abdul et al., 2018), suggesting that aldehyde dehydrogenases play a conserved role in sustaining membrane integrity by scavenging reactive aldehydes, fatty acid radicals, and other alcohol derivatives. All these compounds may be formed during membrane lipid peroxidation, which is triggered mainly by lipoxygenases and reactive oxygen species and is a hallmark of plant pathogen responses (Shah & Chaturvedi, 2008).

The protocatechuate 3,4-dioxygenase beta subunit is another enzyme identified here that participates in the catabolic pathway of protocatechuate (3,4-dihydroxybenzene, PCA); PCA is an aromatic compound and an intermediate product of the degradation of plant biopolymers such as lignin and other aromatic compounds (Brown et al., 2004). Finally, cellobiose dehydrogenase (Scf74_Gene-3.18) is an extracellular flavocytochrome produced by several wood-degrading fungi and appears to be involved in the degradation of both lignin and cellulose by fungi (Ma et al., 2017; Zhang, Fan & Kasuga, 2011). Together, these data highlight an active alcohol metabolic process that implies both lignin degradation in the avocado tissue and detoxification mechanisms to ensure fungal survival. To our knowledge, there is no information regarding the enzymes described above in the *Fusarium* genus.

Another interesting group of proteins that is well known to be involved in fungal pathogenicity is the hydrolytic enzymes. In the case of glycoside hydrolases, an endoglucanase type K (Scf27_Gene-18.41) showed the strongest downregulation at early stages of infection. The UniProt database describes that the catalytic activity of endoglucanase type K is the endohydrolysis of (1-4)-beta-D-glucosidic linkages in cellulose, lichenin and cereal beta-D-glucans; homologs in *F. oxysporum* (OMG_07426) and *F. oxysporum* f. sp. *cubense* tropical race 4 (FOIG_08964) were identified, but no further information was available. Interestingly, during the interaction between avocado and *F. kuroshium*, this protein was repressed; this is not surprising since some plants are capable of secreting inhibitors of certain pathogen hydrolytic enzymes, including glucanases, as part of their defense response (Misas-Villamil & Van der Hoorn, 2008). Therefore, it is possible that this mechanism occurs in avocado. A pectate lyase was also upregulated during the interaction (Scf61_Gene-6.25), and previous evidence clearly demonstrated the importance of this class of enzymes during plant-pathogen interactions since pectin is one of the major components of the plant cell wall. Pectin hydrolysis is a useful invasion or nutrition strategy for phytopathogens. In *Alternaria brassicicola*, a strain with a deletion of the PL1332 gene that encodes a pectate lyase was approximately 30% less virulent than the wild-type in *Brassica oleracea* (Cho et al., 2015). Moreover, purified VdPEL1, a pectate lyase of *Verticillium dahliae*, conferred resistance to *Botrytis cinerea* and *V. dahliae* in tobacco and cotton plants, and the mutant VdPEL1 lacked the ability to induce either cell death or plant resistance, revealing new insights into the role of a pectate lyase during host-pathogen interactions (Yang et al., 2018).

Additionally, proteases, other hydrolytic enzymes, are recognized as powerful tools that are produced by necrotrophic phytopathogens during infection processes; in this context, serine-type carboxypeptidase F (Scf18_Gene-0.42) was upregulated in *F. kuroshium* during the infection process. Serine proteases are hydrolytic enzymes that utilize serine to cleave peptide bonds in proteins. In general, fungi have an extensive repertoire of these enzymes, which are classified into more than 50 families and participate in several cellular activities, including nutrient degradation for subsequent assimilation and protection from the host’s immune system. The type of enzyme we detected in *F. kuroshium* belongs to the S10 superfamily that catalyzes extracellular degradation (Muszewska et al., 2017).

Like previous work in which RNA-seq was performed to document the response of *F. kuroshium* to growth at different pH levels (Sanchez-Rangel et al., 2018), transcripts for proteases, ABC transport and the genes that encode the enzymes involved in FA biosynthesis, a well-documented virulence factor present in other *Fusarium* species (Ding et al., 2018; Liu et al., 2020; López-Díaz et al., 2018), were identified. Interestingly, genes related to FA biosynthesis and another already characterized *Fusarium* toxin were not identified here, possibly because most of the coverage was related to the avocado transcriptome.

However, related to virulence factors, in the fungal gene set, a probable CAP20-virulence factor (Scf71_Gene-7.29) was recorded, although it was completely repressed in all the infection stages in our experiment. It is relevant that *F. kuroshium* is capable of encoding this virulence factor, since studies conducted in *Colletotrichum gloeosporioides* suggested that the perilipin homolog protein involved in the functional appressoria development affects virulence by reducing the penetration of the immature appressoria into the host cuticle (Lin et al., 2018). Moreover, in *F. proliferatum*, secretome profiling was performed during its interaction with banana, and a homolog of the CAP20 virulence factor was identified (CZR38705.1). Because the CAP20 virulence factor may be exclusive to fungi, it is a good candidate for a more detailed study to investigate its participation in *F. kuroshium* infection.

Finally, based on the annotation, 24% of differentially expressed *F. kuroshium* genes were described as uncharacterized proteins, and most of them showed strong upregulation in some of the three infection stages analyzed. It will be interesting to characterize these genes in greater detail in future studies with respect to their function during the infection process.

## Conclusions

Our research was conducted to understand how the vascular tissue of avocado responds during *F. kuroshium* infection, and a deep transcriptome analysis using RNA-Seq revealed a global expression represented in six clusters grouping numerous unigenes related with detoxification and protection mechanisms, regulation pathways such as proteosomes and nitrogen metabolism. Moreover, were detected four genes that encode NLRs involving in immunity and hypersensitivity responses. In counterpart, the alcohol metabolism of *F. kuroshium* is clearly related to its infection process and bring to light that this fungus is capable to induce damage on its own without the presence of the beetle or other symbiont partners such as *G. kuroshium*. For both organisms numerous UniGenes displayed interesting expression patterns with unknown functions, providing numerous potential research options to pursue in the future.

Even though the pathosystem developed control some conditions, such as humidity, temperature, and the conidia suspension concentration, mechanisms such as transport and long-distance signaling, as well as interactions with other organisms and several stresses, are not well represented. Similar experiments could be realized using whole plants to validate both the expression profile of particular genes identified in the present study and identify new resistance and tolerant candidate genes. In addition, and due to the lack of an efficient method to transform avocado plants, we propose the implementation of spray-induced gene silencing (SIGS) or even host-induced gene silencing (HIGS) approaches using composite plants to obtain functional validation of known or unknown *F. kuroshium* responsive genes and even to generate plants resistant to FD disease.

## Supplemental Information

10.7717/peerj.11215/supp-1 Supplemental Information 1 Quality check, data preprocessing, assembly, and avocado (*Persea americana*) transcriptome annotation. (A) Schematic representation of each of the metrics resulting from preprocessing, assembly, and sequence annotation. Wedge size reflects the number of unique sequences (paired-end reads or UniGenes) that were obtained after each step and that were processed and used to obtain the nonredundant sequence set that comprises the avocado coding sequence (CDS) collection. (B) Bar plot showing the number of *Fusarium*-like sequences identified in each of the libraries generated from the infected stem segments. Error bars show the standard deviation from biological replicates (n = 3).

10.7717/peerj.11215/supp-2 Supplemental Information 2 Orthologues detection in different plant species. The shared orthologs, as well as the unique genes identified between compared angiosperm plant species, were identified using OrthoMCL software (Li, Stoeckert & Roos, 2003). Each concentric circle corresponds to one compared species: [1] *Amborella trichopoda*, [2] *Arabidopsis thaliana*, [3] *Musa acuminata* subsp. *malaccensis*, [4] *Persea americana*, [5] *Prunus persica*, [6] *Zea mays*, [7] *Solanum lycopersicum*, and [8] *Vitis vinifera*. Cyan blocks mark the intersection of pairs (orthologs between two species) and show the species-specific genes from each of the species. The height of the purple bars in the outer layer (next to the last concentric circle) corresponds to the number of shared orthologs (x102). To generate this figure, we used the R package SuperExactTest (Wang, Zhao & Zhang, 2015).

10.7717/peerj.11215/supp-3 Supplemental Information 3 Expression profiles associated with the differentially expressed UniGenes involved in the responses to interaction with *F. kuroshium*. Cluster analysis was performed with k-means methods (n = 6) using Genesis software (Sturn, Quackenbush & Trajanoski, 2002). The x-axis represents the days after inoculation (1-dpi, 4-dpi, 7-dpi, and 14-dpi, respectively). The y-axis represents the Log2 fold-change values calculated once infected and uninfected avocado stems-segments were compared. Gray lines, expression ofsingle genes trough the time; pink line, average of all genes in cluster. On the right side of k-means cluster graphs, a Heatmap of the differentially expressed UniGenes and contained in each cluster. Red, blue, and white indicate upregulation, downregulation, and no change, once infected and uninfected stems-segments were compared at 1, 4, 7 and 14 dpi. Each column represents the data from one day. The list of genes grouped in each cluster is shown in Table S6.

10.7717/peerj.11215/supp-4 Supplemental Information 4 Schematic representation of the motifs contained in the N-terminal domain of the avocado CC-type NLR proteins. (A) The consensus sequence pattern of the MADA motif, which was identified in a total of 30 avocado CC-NLR proteins. Red boxes refer to residues that were highly conserved and previously reported in plant species (Adachi et al., 2019). (B) Three clearly distinguishable major groups (A, B and C) were identified after aligning and resolving their phylogenetic relationships based only on the CC domain, which contains a conserved array of amino acids known as the EDVID motif or the EDVID-corresponding area (Wróblewski et al., 2018). WebLogo (Crooks et al., 2004) with small-sample correction (Schneider et al., 1986) was used to generate the graphical representations of both domains (N-terminal domain CC-type and NBS, respectively).

10.7717/peerj.11215/supp-5 Supplemental Information 5 Schematic representation of the motifs contained in the NBS domain of the avocado CC-type NLR proteins. Schematic representation of the motifs contained in the NBS domain of the avocado CC-type NLR proteins. The consensus sequence of each of the highly conserved motifs in the NBS domain (P-loop, kinase, RNBS, GLPL, MHD; (Van Ghelder et al., 2019) was graphically displayed with WebLogo (Crooks et al., 2004) using small-sample correction (Schneider et al., 1986).

10.7717/peerj.11215/supp-6 Supplemental Information 6 3D structure models of a putative avocado resistosome. From left to right, the figure shows the side, top, and bottom views of the structure conformed by five monomers, all of them corresponding either to UN001791 (A) or UN003288 (B), respectively. These UniGenes encode avocado NLR receptors, both orthologs of the ZAR1 *A. thaliana* protein. To show these structures in their activated form, the ZAR1-RKS1-PBL21 resistosome [6j5t.pdb; (Wang et al., 2019a) was used as a template. In the modeling, no orthologs to RKS1 or PBL2 from avocado were included. The characteristic consensus sequence pattern of the MADA motif and its specific sequences in UN001791 and UN003288 are also shown (C) and are highlighted by red boxes in the putative resistosome structures.

10.7717/peerj.11215/supp-7 Supplemental Information 7 Tables S1-S5.

10.7717/peerj.11215/supp-8 Supplemental Information 8 Tables S6-S14.

10.7717/peerj.11215/supp-9 Supplemental Information 9 Protein sequences.

## Additional Information and Declarations

### Data Availability

The following information was supplied regarding data availability: The data are available in the Short Read Archive (SRA) of the National Center for Biotechnology Information (NCBI): PRJNA655460.

## References

[ref-1] Abbasi PA, Graham MY, Graham TL (2001). Effects of soybean genotype on the glyceollin elicitation competency of cotyledon tissues to *Phytophthora sojae* glucan elicitors. Physiological and Molecular Plant Pathology.

[ref-2] Abdul W, Aliyu SR, Lin L, Sekete M, Chen X, Otieno FJ, Yang T, Lin Y, Norvienyeku J, Wang Z (2018). Family-four aldehyde dehydrogenases play an indispensable role in the pathogenesis of *Magnaporthe oryzae*. Frontiers in Plant Science.

[ref-3] Adachi H, Contreras MP, Harant A, Wu CH, Derevnina L, Sakai T, Duggan C, Moratto E, Bozkurt TO, Maqbool A, Win J, Kamoun S (2019). An N-terminal motif in NLR immune receptors is functionally conserved across distantly related plant species. eLife.

[ref-4] Adachi H, Derevnina L, Kamoun S (2019). NLR singletons, pairs, and networks: evolution, assembly, and regulation of the intracellular immunoreceptor circuitry of plants. Current Opinion in Plant Biology.

[ref-5] Ali SS, Gunupuru LR, Kumar GBS, Khan M, Scofield S, Nicholson P, Doohan FM (2014). Plant disease resistance is augmented in uzu barley lines modified in the brassinosteroid receptor BRI1. BMC Plant Biology.

[ref-6] Altschul SF, Gish W, Miller W, Myers EW, Lipman DJ (1990). Basic local alignment search tool. Journal of Molecular Biology.

[ref-7] Anders S, Huber W (2010). Differential expression analysis for sequence count data. Genome Biology.

[ref-8] Anisimova M, Gascuel O (2006). Approximate likelihood-ratio test for branches: a fast, accurate, and powerful alternative. Systematic Biology.

[ref-9] Arnold K, Bordoli L, Kopp J, Schwede T (2006). The SWISS-MODEL workspace: a web-based environment for protein structure homology modelling. Bioinformatics.

[ref-10] Ashburner M, Ball CA, Blake JA, Botstein D, Butler H, Cherry JM, Davis AP, Dolinski K, Dwight SS, Eppig JT, Harris MA, Hill DP, Issel-Tarver L, Kasarskis A, Lewis S, Matese JC, Richardson JE, Ringwald M, Rubin GM, Sherlock G (2000). Gene ontology: tool for the unification of biology. Nature Genetics.

[ref-11] Benjamini Y, Hochberg Y (1995). Controlling the false discovery rate: a practical and powerful approach to multiple testing. Journal of the Royal Statistical Society Series B.

[ref-12] Bentham AR, Zdrzalek R, De la Concepcion JC, Banfield MJ (2018). Uncoiling CNLs: structure/function approaches to understanding CC domain function in plant NLRs. Plant and Cell Physiology.

[ref-13] Björklund S, Gustafsson CM (2005). The yeast Mediator complex and its regulation. Trends in Biochemical Sciences.

[ref-14] Boutrot F, Zipfel C (2017). Function, discovery, and exploitation of plant pattern recognition receptors for broad-spectrum disease resistance. Annual Review of Phytopathology.

[ref-15] Bozsoki Z, Gysel K, Hansen SB, Lironi D, Krönauer C, Feng F, De Jong N, Vinther M, Kamble M, Thygesen MB, Engholm E, Kofoed C, Fort S, Sullivan JT, Ronson CW, Jensen KJ, Blaise M, Oldroyd G, Stougaard J, Andersen KR, Radutoiu S (2020). Ligand-recognizing motifs in plant LysM receptors are major determinants of specificity. Science.

[ref-16] Brown CK, Vetting MW, Earhart CA, Ohlendorf DH (2004). Biophysical analyses of designed and selected mutants of protocatechuate 3,4-dioxygenase1. Annual Review of Microbiology.

[ref-17] Burdett H, Bentham AR, Williams SJ, Dodds PN, Anderson PA, Banfield MJ, Kobe B (2019). The plant “resistosome”: structural insights into immune signaling. Cell Host & Microbe.

[ref-18] Carrillo D, Cruz LF, Kendra PE, Narvaez TI, Montgomery WS, Monterroso A, De Grave C, Cooperband MF (2016). Distribution, pest status and fungal associates of *Euwallacea* nr. *fornicatus* in Florida avocado groves. Insects.

[ref-19] Carrillo JD, Mayorquin JS, Stajich JE, Eskalen A (2019). Probe-based multiplex real-time PCR as a diagnostic tool to distinguish distinct fungal symbionts associated with *Euwallacea kuroshio* and *E. whitfordiodendrus* in California. Plant Disease.

[ref-20] Cass CL, Peraldi A, Dowd PF, Mottiar Y, Santoro N, Karlen SD, Bukhman YV, Foster CE, Thrower N, Bruno LC, Moskvin OV, Johnson ET, Willhoit ME, Phutane M, Ralph J, Mansfield SD, Nicholson P, Sedbrook JC (2015). Effects of PHENYLALANINE AMMONIA LYASE (PAL) knockdown on cell wall composition, biomass digestibility, and biotic and abiotic stress responses in *Brachypodium*. Journal of Experimental Botany.

[ref-21] Chang CS, Li YH, Chen LT, Chen WC, Hsieh WP, Shin J, Jane WN, Chou SJ, Choi G, Hu JM, Somerville S, Wu SH (2008). LZF1, a HY5-regulated transcriptional factor, functions in *Arabidopsis* de-etiolation. Plant Journal.

[ref-22] Cho Y, Jang M, Srivastava A, Jang JH, Soung NK, Ko SK, Kang DO, Ahn JS, Kim BY (2015). A pectate lyase-coding gene abundantly expressed during early stages of infection is required for full virulence in *Alternaria brassicicola*. PLOS ONE.

[ref-23] Collier SM, Hamel LP, Moffett P (2011). Cell death mediated by the N-terminal domains of a unique and highly conserved class of NB-LRR protein. Molecular Plant-Microbe Interactions®.

[ref-24] Couderc R, Baratti J (1980). Oxidation of methanol by the yeast, *Pichia pastoris*. Purification and properties of the alcohol oxidase. Agricultural and Biological Chemistry.

[ref-25] Cregg JM, Madden KR, Barringer KJ, Thill GP, Stillman CA (1989). Functional characterization of the two alcohol oxidase genes from the yeast *Pichia pastoris*. Molecular and Cellular Biology.

[ref-26] Crooks GE, Hon G, Chandonia JM, Brenner SE (2004). WebLogo: a sequence logo generator. Genome Research.

[ref-27] Danot O, Marquenet E, Vidal-Ingigliardi D, Richet E (2009). Wheel of life, wheel of death: a mechanistic insight into signaling by STAND proteins. Structure.

[ref-28] Dauphinee AN, Fletcher JI, Denbigh GL, Lacroix CR, Gunawardena A (2017). Remodelling of lace plant leaves: antioxidants and ROS are key regulators of programmed cell death. Planta.

[ref-29] Deng Y, Zhai K, Xie Z, Yang D, Zhu X, Liu J, Wang X, Qin P, Yang Y, Zhang G, Li Q, Zhang J, Wu S, Milazzo J, Mao B, Wang E, Xie H, Tharreau D, He Z (2017). Epigenetic regulation of antagonistic receptors confers rice blast resistance with yield balance. Science.

[ref-30] Di Matteo A, Federici L, Mattei B, Salvi G, Johnson KA, Savino C, De Lorenzo G, Tsernoglou D, Cervone F (2003). The crystal structure of polygalacturonase-inhibiting protein (PGIP), a leucine-rich repeat protein involved in plant defense. Proceedings of the National Academy of Sciences of the United States of America.

[ref-31] Ding Z, Yang L, Wang G, Guo L, Liu L, Wang J, Huang J (2018). Fusaric acid is a virulence factor of *Fusarium oxysporum* f. sp. *cubense* on banana plantlets. Tropical Plant Pathology.

[ref-32] Edgar RC (2004). MUSCLE: multiple sequence alignment with high accuracy and high throughput. Nucleic Acids Research.

[ref-33] Enright AJ, Van Dongen S, Ouzounis CA (2002). An efficient algorithm for large-scale detection of protein families. Nucleic Acids Research.

[ref-34] Ero R, Kumar V, Su W, Gao Y-G (2019). Ribosome protection by ABC-F proteins-Molecular mechanism and potential drug design. Protein Science : A Publication of the Protein Society.

[ref-35] Eskalen A, Gonzalez A, Wang DH, Twizeyimana M, Mayorquin JS, Lynch SC (2012). First report of a *Fusarium* sp. and its vector tea shot hole borer (*Euwallacea fornicatus*) causing Fusarium Dieback on avocado in California. Plant Disease.

[ref-36] Eskalen A, Stouthamer R, Lynch SC, Rugman-Jones PF, Twizeyimana M, Gonzalez A, Thibault T (2013). Host range of Fusarium Dieback and its ambrosia beetle (Coleoptera: Scolytinae) vector in southern California. Plant Disease.

[ref-37] Evans T, Loose M (2015). AlignWise: a tool for identifying protein-coding sequence and correcting frame-shifts. BMC Bioinformatics.

[ref-38] Finn RD, Coggill P, Eberhardt RY, Eddy SR, Mistry J, Mitchell AL, Potter SC, Punta M, Qureshi M, Sangrador-Vegas A, Salazar GA, Tate J, Bateman A (2016). The Pfam protein families database: towards a more sustainable future. Nucleic Acids Research.

[ref-39] Freeman S, Sharon M, Dori-Bachash M, Maymon M, Belausov E, Maoz Y, Margalit O, Protasov A, Mendel Z (2016). Symbiotic association of three fungal species throughout the life cycle of the ambrosia beetle *Euwallacea* nr. *fornicatus*. Symbiosis.

[ref-40] Freeman S, Sharon M, Maymon M, Mendel Z, Protasov A, Aoki T, Eskalen A, O’Donnell K (2013). *Fusarium euwallaceae* sp. nov.—a symbiotic fungus of *Euwallacea* sp., an invasive ambrosia beetle in Israel and California. Mycologia.

[ref-41] Fu ZQ, Dong X (2013). Systemic acquired resistance: turning local infection into global defense. Annual Review of Plant Biology.

[ref-42] García-Avila CDJ, Trujillo-Arriaga FJ, López-Buenfil JA, González-Gómez R, Carrillo D, Cruz LF, Ruiz-Galván I, Quezada-Salinas A, Acevedo-Reyes N (2016). First report of *Euwallacea* nr. *fornicatus* (Coleoptera: Curculionidae) in Mexico. Florida Entomologist.

[ref-43] Gascuel O (1997). BIONJ: an improved version of the NJ algorithm based on a simple model of sequence data. Molecular Biology and Evolution.

[ref-44] Godoy AV, Lazzaro AS, Casalongué CA, San Segundo B (2000). Expression of a *Solanum tuberosum* cyclophilin gene is regulated by fungal infection and abiotic stress conditions. Plant Science.

[ref-45] Gomez DF, Skelton J, Steininger MS, Stouthamer R, Rugman-Jones P, Sittichaya W, Rabaglia RJ, Hulcr J (2018). Species delineation within the *Euwallacea fornicatus* (Coleoptera: Curculionidae) complex revealed by morphometric and phylogenetic analyses. Insect Systematics and Diversity.

[ref-46] Goodman CD, Casati P, Walbot V (2004). A multidrug resistance-associated protein involved in anthocyanin transport in *Zea mays*. Plant Cell.

[ref-47] Gouy M, Guindon S, Gascuel O (2010). SeaView version 4: a multiplatform graphical user interface for sequence alignment and phylogenetic tree building. Molecular Biology and Evolution.

[ref-48] Grabherr MG, Haas BJ, Yassour M, Levin JZ, Thompson DA, Amit I, Adiconis X, Fan L, Raychowdhury R, Zeng Q, Chen Z, Mauceli E, Hacohen N, Gnirke A, Rhind N, Di Palma F, Birren BW, Nusbaum C, Lindblad-Toh K, Friedman N, Regev A (2011). Full-length transcriptome assembly from RNA-Seq data without a reference genome. Nature Biotechnology.

[ref-49] Guindon S, Dufayard JF, Lefort V, Anisimova M, Hordijk W, Gascuel O (2010). New algorithms and methods to estimate maximum-likelihood phylogenies: assessing the performance of PhyML 3.0. Systematic Biology.

[ref-50] Guindon S, Gascuel O (2003). A simple, fast, and accurate algorithm to estimate large phylogenies by maximum likelihood. Systematic Biology.

[ref-51] He S, Yuan G, Bian S, Han X, Liu K, Cong P, Zhang C (2020). Major latex protein MdMLP423 negatively regulates defense against fungal infections in apple. International Journal of Molecular Sciences.

[ref-52] He Z, Li L, Luan S (2004). Immunophilins and parvulins. Superfamily of peptidyl prolyl isomerases in *Arabidopsis*. Plant Physiology.

[ref-53] Hulcr J, Stelinski LL (2017). The ambrosia symbiosis: from evolutionary ecology to practical management. Annual Review of Entomology.

[ref-54] Hwang IS, An SH, Hwang BK (2011). Pepper asparagine synthetase 1 (CaAS1) is required for plant nitrogen assimilation and defense responses to microbial pathogens. Plant Journal.

[ref-55] Ibarra-Laclette E, Sánchez-Rangel D, Hernández-Domínguez E, Pérez-Torres CA, Ortiz-Castro R, Villafán E, Alonso-Sánchez A, Rodríguez-Hass B, López-Buenfil A, García-Avila C, Ramírez-Pool JA (2017). Draft genome sequence of the phytopathogenic fungus *Fusarium euwallaceae*, the causal agent of Fusarium Dieback. Genome Announcements.

[ref-56] Inch S, Ploetz R, Held B, Blanchette R (2012). Histological and anatomical responses in avocado, *Persea americana*, induced by the vascular wilt pathogen, *Raffaelea lauricola*. Botany.

[ref-57] Jones JD, Vance RE, Dangl JL (2016). Intracellular innate immune surveillance devices in plants and animals. Science.

[ref-58] Kanehisa M, Sato Y, Kawashima M, Furumichi M, Tanabe M (2016). KEGG as a reference resource for gene and protein annotation. Nucleic Acids Research.

[ref-59] Kang J, Park J, Choi H, Burla B, Kretzschmar T, Lee Y, Martinoia E (2011). Plant ABC transporters. Arabidopsis Book.

[ref-60] Karpinski S, Reynolds H, Karpinska B, Wingsle G, Creissen G, Mullineaux P (1999). Systemic signaling and acclimation in response to excess excitation energy in *Arabidopsis*. Science.

[ref-61] Kebede AZ, Johnston A, Schneiderman D, Bosnich W, Harris LJ (2018). Transcriptome profiling of two maize inbreds with distinct responses to Gibberella ear rot disease to identify candidate resistance genes. BMC Genomics.

[ref-62] Khanna R, Kronmiller B, Maszle DR, Coupland G, Holm M, Mizuno T, Wu S-H (2009). The *Arabidopsis* B-box zinc finger family. Plant Cell.

[ref-63] Kim D, Langmead B, Salzberg SL (2015). HISAT: a fast spliced aligner with low memory requirements. Nature Methods.

[ref-64] Kim DS, Hwang BK (2014). An important role of the pepper phenylalanine ammonia-lyase gene (PAL1) in salicylic acid-dependent signalling of the defence response to microbial pathogens. Journal of Experimental Botany.

[ref-65] Kim JH, Castroverde CDM (2020). Diversity, function and regulation of cell surface and intracellular immune receptors in Solanaceae. Plants.

[ref-66] Klein M, Burla B, Martinoia E (2006). The multidrug resistance-associated protein (MRP/ABCC) subfamily of ATP-binding cassette transporters in plants. FEBS Letters.

[ref-67] Koch C, Neumann P, Valerius O, Feussner I, Ficner R (2016). Crystal structure of alcohol oxidase from *Pichia pastoris*. PLOS ONE.

[ref-68] Kornberg RD (2005). Mediator and the mechanism of transcriptional activation. Trends in Biochemical Sciences.

[ref-69] Langmead B, Salzberg SL (2012). Fast gapped-read alignment with Bowtie 2. Nature Methods.

[ref-70] Levin DA (1973). The role of trichomes in plant defense. Quarterly Review of Biology.

[ref-71] Lewis JD, Lee AH, Hassan JA, Wan J, Hurley B, Jhingree JR, Wang PW, Lo T, Youn JY, Guttman DS, Desveaux D (2013). The *Arabidopsis* ZED1 pseudokinase is required for ZAR1-mediated immunity induced by the *Pseudomonas syringae* type III effector HopZ1a. Proceedings of the National Academy of Sciences of the United States of America.

[ref-72] Li B, Dewey CN (2011). RSEM: accurate transcript quantification from RNA-Seq data with or without a reference genome. BMC Bioinformatics.

[ref-73] Li L, Stoeckert CJ, Roos DS (2003). OrthoMCL: identification of ortholog groups for eukaryotic genomes. Genome Research.

[ref-74] Li X, Zhong S, Chen W, Fatima SA, Huang Q, Li Q, Tan F, Luo P (2018). Transcriptome analysis identifies a 140 kb region of chromosome 3B containing genes specific to Fusarium head blight resistance in wheat. International Journal of Molecular Sciences.

[ref-75] Li Y-J, Zhu S-H, Zhang X-Y, Liu Y-C, Xue F, Zhao L-J, Sun J (2017). Expression and functional analyses of a Kinesin gene GhKIS13A1 from cotton (*Gossypium hirsutum*) fiber. BMC Biotechnology.

[ref-76] Lin C, Liu X, Shi T, Li C, Huang G (2018). The *Colletotrichum gloeosporioides* perilipin homologue CAP 20 regulates functional appressorial formation and fungal virulence. Journal of Phytopathology.

[ref-77] Lin Y, Zou W, Lin S, Onofua D, Yang Z, Chen H, Wang S, Chen X (2017). Transcriptome profiling and digital gene expression analysis of sweet potato for the identification of putative genes involved in the defense response against *Fusarium oxysporum* f. sp. batatas. PLOS ONE.

[ref-78] Liu S, Li J, Zhang Y, Liu N, Viljoen A, Mostert D, Zuo C, Hu C, Bi F, Gao H, Sheng O, Deng G, Yang Q, Dong T, Dou T, Yi G, Ma LJ, Li C (2020). Fusaric acid instigates the invasion of banana by *Fusarium oxysporum* f. sp. *cubense* TR4. New Phytologist.

[ref-79] López-Díaz C, Rahjoo V, Sulyok M, Ghionna V, Martín-Vicente A, Capilla J, Di Pietro A, López-Berges MS (2018). Fusaric acid contributes to virulence of *Fusarium oxysporum* on plant and mammalian hosts. Molecular Plant Pathology.

[ref-80] Lu L, Lee YR, Pan R, Maloof JN, Liu B (2005). An internal motor kinesin is associated with the Golgi apparatus and plays a role in trichome morphogenesis in *Arabidopsis*. Molecular Biology of the Cell.

[ref-81] Lukasik E, Takken FL (2009). STANDing strong, resistance proteins instigators of plant defence. Current Opinion in Plant Biology.

[ref-82] Lynch SC, Twizeyimana M, Mayorquin JS, Wang DH, Na F, Kayim M, Kasson MT, Thu PQ, Bateman C, Rugman-Jones P, Hulcr J, Stouthamer R, Eskalen A (2016). Identification, pathogenicity and abundance of *Paracremonium pembeum* sp. nov. and *Graphium euwallaceae* sp. nov.—two newly discovered mycangial associates of the polyphagous shot hole borer (*Euwallacea* sp.) in California. Mycologia.

[ref-84] Ma LJ, Geiser DM, Proctor RH, Rooney AP, O’Donnell K, Trail F, Gardiner DM, Manners JM, Kazan K (2013). *Fusarium* pathogenomics. Annual Review of Microbiology.

[ref-85] Ma S, Preims M, Piumi F, Kappel L, Seiboth B, Record E, Kracher D, Ludwig R (2017). Molecular and catalytic properties of fungal extracellular cellobiose dehydrogenase produced in prokaryotic and eukaryotic expression systems. Microbial Cell Factories.

[ref-86] Marchler-Bauer A, Lu S, Anderson JB, Chitsaz F, Derbyshire MK, DeWeese-Scott C, Fong JH, Geer LY, Geer RC, Gonzales NR, Gwadz M, Hurwitz DI, Jackson JD, Ke Z, Lanczycki CJ, Lu F, Marchler GH, Mullokandov M, Omelchenko MV, Robertson CL, Song JS, Thanki N, Yamashita RA, Zhang D, Zhang N, Zheng C, Bryant SH (2011). CDD: a Conserved Domain Database for the functional annotation of proteins. Nucleic Acids Research.

[ref-87] Mendel Z, Protasov A, Sharon M, Zveibil A, Yehuda SB, O’Donnell K, Rabaglia R, Wysoki M, Freeman S (2012). An Asian ambrosia beetle *Euwallacea fornicatus* and its novel symbiotic fungus *Fusarium* sp. pose a serious threat to the Israeli avocado industry. Phytoparasitica.

[ref-88] Meyers BC, Kozik A, Griego A, Kuang H, Michelmore RW (2003). Genome-wide analysis of NBS-LRR-encoding genes in *Arabidopsis*. Plant Cell.

[ref-89] Miricescu A, Goslin K, Graciet E (2018). Ubiquitylation in plants: signaling hub for the integration of environmental signals. Journal of Experimental Botany.

[ref-90] Misas-Villamil JC, Van der Hoorn RAL (2008). Enzyme-inhibitor interactions at the plant-pathogen interface. Current Opinion in Plant Biology.

[ref-91] Moffett P, Farnham G, Peart J, Baulcombe DC (2002). Interaction between domains of a plant NBS-LRR protein in disease resistance-related cell death. EMBO Journal.

[ref-92] Monteiro F, Nishimura MT (2018). Structural, functional, and genomic diversity of plant NLR proteins: an evolved resource for rational engineering of plant immunity. Annual Review of Phytopathology.

[ref-93] Muszewska A, Stepniewska-Dziubinska MM, Steczkiewicz K, Pawlowska J, Dziedzic A, Ginalski K (2017). Fungal lifestyle reflected in serine protease repertoire. Scientific Reports.

[ref-94] Na F, Carrillo JD, Mayorquin JS, Ndinga-Muniania C, Stajich JE, Stouthamer R, Huang Y-T, Lin Y-T, Chen CY, Eskalen A (2017). Two novel fungal symbionts *Fusarium kuroshium* sp. nov. and *Graphium kuroshium* sp. nov. of Kuroshio shot hole borer (*Euwallacea sp.* nr. *fornicatus*) cause Fusarium dieback on woody host species in California. Plant Disease.

[ref-95] Nürnberger T, Kemmerling B (2006). Receptor protein kinases: pattern recognition receptors in plant immunity. Trends in Plant Science.

[ref-96] Ohtake Y, Takahashi T, Komeda Y (2000). Salicylic acid induces the expression of a number of receptor-like kinase genes in *Arabidopsis thaliana*. Plant and Cell Physiology.

[ref-97] Pan Y, Liu Z, Rocheleau H, Fauteux F, Wang Y, McCartney C, Ouellet T (2018). Transcriptome dynamics associated with resistance and susceptibility against *Fusarium* head blight in four wheat genotypes. BMC Genomics.

[ref-98] Petricka JJ, Nelson TM (2007). *Arabidopsis* nucleolin affects plant development and patterning. Plant Physiology.

[ref-99] Pogorelko GV, Mokryakova M, Fursova OV, Abdeeva I, Piruzian ES, Bruskin SA (2014). Characterization of three *Arabidopsis thaliana* immunophilin genes involved in the plant defense response against *Pseudomonas syringae*. Gene.

[ref-100] Punta M, Coggill PC, Eberhardt RY, Mistry J, Tate J, Boursnell C, Pang N, Forslund K, Ceric G, Clements J, Heger A, Holm L, Sonnhammer ELL, Eddy SR, Bateman A, Finn RD (2012). The Pfam protein families database. Nucleic Acids Research.

[ref-101] Ranf S, Gisch N, Schäffer M, Illig T, Westphal L, Knirel YA, Sánchez-Carballo PM, Zähringer U, Hückelhoven R, Lee J, Scheel D (2015). A lectin S-domain receptor kinase mediates lipopolysaccharide sensing in *Arabidopsis thaliana*. Nature Immunology.

[ref-102] Raudvere U, Kolberg L, Kuzmin I, Arak T, Adler P, Peterson H, Vilo J (2019). g: Profiler: a web server for functional enrichment analysis and conversions of gene lists (2019 update). Nucleic Acids Research.

[ref-103] Rauwane ME, Ogugua UV, Kalu CM, Ledwaba LK, Woldesemayat AA, Ntushelo K (2020). Pathogenicity and virulence factors of *Fusarium graminearum* including factors discovered using next generation sequencing technologies and proteomics. Microorganisms.

[ref-104] Rea PA (2007). Plant ATP-binding cassette transporters. Annual Review of Plant Biology.

[ref-105] Rendon-Anaya M, Ibarra-Laclette E, Mendez-Bravo A, Lan T, Zheng C, Carretero-Paulet L, Perez-Torres CA, Chacon-Lopez A, Hernandez-Guzman G, Chang TH, Farr KM, Barbazuk WB, Chamala S, Mutwil M, Shivhare D, Alvarez-Ponce D, Mitter N, Hayward A, Fletcher S, Rozas J, Sanchez Gracia A, Kuhn D, Barrientos-Priego AF, Salojarvi J, Librado P, Sankoff D, Herrera-Estrella A, Albert VA, Herrera-Estrella L (2019). The avocado genome informs deep angiosperm phylogeny, highlights introgressive hybridization, and reveals pathogen-influenced gene space adaptation. Proceedings of the National Academy of Sciences of the United States of America.

[ref-106] Romano PG, Horton P, Gray JE (2004). The *Arabidopsis* cyclophilin gene family. Plant Physiology.

[ref-107] Sahu BB, Baumbach JL, Singh P, Srivastava SK, Yi X, Bhattacharyya MK (2017). Investigation of the *Fusarium virguliforme* transcriptomes induced during infection of soybean roots suggests that enzymes with hydrolytic activities could play a major role in root necrosis. PLOS ONE.

[ref-108] Sanchez-Rangel D, Hernandez-Dominguez EE, Perez-Torres CA, Ortiz-Castro R, Villafan E, Rodriguez-Haas B, Alonso-Sanchez A, Lopez-Buenfil A, Carrillo-Ortiz N, Hernandez-Ramos L, Ibarra-Laclette E (2018). Environmental pH modulates transcriptomic responses in the fungus *Fusarium* sp. associated with KSHB *Euwallacea* sp. near *fornicatus*. BMC Genomics.

[ref-109] Shiu SH, Bleecker AB (2003). Expansion of the receptor-like kinase/Pelle gene family and receptor-like proteins in *Arabidopsis*. Plant Physiology.

[ref-110] Schneider TD, Stormo GD, Gold L, Ehrenfeucht A (1986). Information content of binding sites on nucleotide sequences. Journal of Molecular Biology.

[ref-111] Segers G, Bradshaw N, Archer D, Blissett K, Oliver RP (2001). Alcohol oxidase is a novel pathogenicity factor for *Cladosporium fulvum*, but aldehyde dehydrogenase is dispensable. Molecular Plant-Microbe Interactions®.

[ref-112] Seifi H, De Vleesschauwer D, Aziz A, Höfte M (2014). Modulating plant primary amino acid metabolism as a necrotrophic virulence strategy: the immune-regulatory role of asparagine synthetase in *Botrytis cinerea*-tomato interaction. Plant Signaling & Behavior.

[ref-151] SENASICA (2019). https://prod.senasica.gob.mx/SIRVEF/ContenidoPublico/Fichas%20tecnicas/Ficha%20T%C3%A9cnica%20del%20escarabajo%20barrenador%20pol%C3%ADfago.pdf.

[ref-113] Seto D, Koulena N, Lo T, Menna A, Guttman DS, Desveaux D (2017). Expanded type III effector recognition by the ZAR1 NLR protein using ZED1-related kinases. Nature Plants.

[ref-114] Shah J, Chaturvedi R (2008). Lipid signals in plant-pathogen interactions. Molecular Aspects of Plant Disease Resistance.

[ref-115] Silvia Sebastiani M, Bagnaresi P, Sestili S, Biselli C, Zechini A, Orru L, Cattivelli L, Ficcadenti N (2017). Transcriptome analysis of the melon-*Fusarium oxysporum* f. sp. *melonis* Race 1.2 pathosystem in susceptible and resistant plants. Frontiers in Plant Science.

[ref-116] Simao FA, Waterhouse RM, Ioannidis P, Kriventseva EV, Zdobnov EM (2015). BUSCO: assessing genome assembly and annotation completeness with single-copy orthologs. Bioinformatics.

[ref-117] Six DL, Wingfield MJ (2011). The role of phytopathogenicity in bark beetle-fungus symbioses: a challenge to the classic paradigm. Annual Review of Entomology.

[ref-83] Smith SM, Gomez DF, Beaver RA, Hulcr J, Cognato AI (2019). Reassessment of the species in the *Euwallacea fornicatus* (Coleoptera: Curculionidae: Scolytinae) complex after the rediscovery of the “lost” type specimen. Insects.

[ref-118] Sonawala U, Dinkeloo K, Danna CH, McDowell JM, Pilot G (2018). Review: Functional linkages between amino acid transporters and plant responses to pathogens. Plant Science.

[ref-119] Srinivas C, Nirmala Devi D, Narasimha Murthy K, Mohan CD, Lakshmeesha TR, Singh B, Kalagatur NK, Niranjana SR, Hashem A, Alqarawi AA, Tabassum B, Abd Allah EF, Chandra Nayaka S (2019). *Fusarium oxysporum* f. sp. *lycopersici* causal agent of vascular wilt disease of tomato: biology to diversity—a review. Saudi Journal of Biological Sciences.

[ref-120] Steele JFC, Hughes RK, Banfield MJ (2019). Structural and biochemical studies of an NB-ARC domain from a plant NLR immune receptor. PLOS ONE.

[ref-121] Sturn A, Quackenbush J, Trajanoski Z (2002). Genesis: cluster analysis of microarray data. Bioinformatics.

[ref-122] Supek F, Bosnjak M, Skunca N, Smuc T (2011). REVIGO summarizes and visualizes long lists of gene ontology terms. PLOS ONE.

[ref-123] Teixeira MA, Rajewski A, He J, Castaneda OG, Litt A, Kaloshian I (2018). Classification and phylogenetic analyses of the *Arabidopsis* and tomato G-type lectin receptor kinases. BMC Genomics.

[ref-124] Van Ghelder C, Parent GJ, Rigault P, Prunier J, Giguère I, Caron S, Stival Sena J, Deslauriers A, Bousquet J, Esmenjaud D, MacKay J (2019). The large repertoire of conifer NLR resistance genes includes drought responsive and highly diversified RNLs. Scientific Reports.

[ref-125] Van Ooijen G, Mayr G, Kasiem MMA, Albrecht M, Cornelissen BJC, Takken FLW (2008). Structure-function analysis of the NB-ARC domain of plant disease resistance proteins. Journal of Experimental Botany.

[ref-126] Van Wersch S, Tian L, Hoy R, Li X (2020). Plant NLRs: the whistleblowers of plant immunity. Plant Communications.

[ref-127] Vázquez-Rosas-Landa M, Sánchez-Rangel D, Hernández-Domínguez EE, Pérez-Torres CA, López-Buenfil A, Garcia-Avila CDJ, Carrillo-Hernández ED, Castañeda-Casasola CC, Rodríguez-Haas B, Pérez-Lira J, Villafan E, Alonso-Sanchez A, Ibarra-Laclette E (2021). Design of a diagnostic system based on molecular markers derived from the Ascomycetes pan-genome analysis: the case of Fusarium Dieback disease. PLOS ONE.

[ref-128] Veronica De M, Angela B, Elisabeth AW, Pieter B (2016). Tyloses and gums: a review of structure, function, and occurrence of vessel occlusions. IAWA Journal.

[ref-129] Visser EA, Wegrzyn JL, Myburg AA, Naidoo S (2018). Defence transcriptome assembly and pathogenesis related gene family analysis in *Pinus tecunumanii* (low elevation). BMC Genomics.

[ref-130] Wang G, Ellendorff U, Kemp B, Mansfield JW, Forsyth A, Mitchell K, Bastas K, Liu CM, Woods-Tör A, Zipfel C, De Wit PJ, Jones JD, Tör M, Thomma BP (2008). A genome-wide functional investigation into the roles of receptor-like proteins in *Arabidopsis*. Plant Physiology.

[ref-131] Wang X, Zafian P, Choudhary M, Lawton M (1996). The PR5K receptor protein kinase from *Arabidopsis thaliana* is structurally related to a family of plant defense proteins. Proceedings of the National Academy of Sciences of the United States of America.

[ref-132] Wan Y, King R, Mitchell RAC, Hassani-Pak K, Hawkesford MJ (2017). Spatiotemporal expression patterns of wheat amino acid transporters reveal their putative roles in nitrogen transport and responses to abiotic stress. Scientific Reports.

[ref-133] Wang J, Hu M, Wang J, Qi J, Han Z, Wang G, Qi Y, Wang HW, Zhou JM, Chai J (2019a). Reconstitution and structure of a plant NLR resistosome conferring immunity. Science.

[ref-134] Wang J, Wang J, Hu M, Wu S, Qi J, Wang G, Han Z, Qi Y, Gao N, Wang HW, Zhou JM, Chai J (2019b). Ligand-triggered allosteric ADP release primes a plant NLR complex. Science.

[ref-135] Wang L, Li Q, Liu Z, Surendra A, Pan Y, Li Y, Zaharia LI, Ouellet T, Fobert PR (2018). Integrated transcriptome and hormone profiling highlight the role of multiple phytohormone pathways in wheat resistance against *Fusarium* head blight. PLOS ONE.

[ref-136] Wang M, Zhao Y, Zhang B (2015). Efficient test and visualization of multi-set intersections. Scientific Reports.

[ref-137] Wang Y, Yang L, Chen X, Ye T, Zhong B, Liu R, Wu Y, Chan Z (2015). Major latex protein-like protein 43 (MLP43) functions as a positive regulator during abscisic acid responses and confers drought tolerance in Arabidopsis thaliana. Journal of Experimental Botany.

[ref-138] Wang Y, Zhou Z, Gao J, Wu Y, Xia Z, Zhang H, Wu J (2016). The mechanisms of maize resistance to *Fusarium verticillioides* by comprehensive analysis of RNA-seq data. Frontiers in Plant Science.

[ref-139] Wathugala DL, Hemsley PA, Moffat CS, Cremelie P, Knight MR, Knight H (2012). The Mediator subunit SFR6/MED16 controls defence gene expression mediated by salicylic acid and jasmonate responsive pathways. New Phytologist.

[ref-140] Wróblewski T, Spiridon L, Martin EC, Petrescu AJ, Cavanaugh K, Truco MJ, Xu H, Gozdowski D, Pawłowski K, Michelmore RW, Takken FLW (2018). Genome-wide functional analyses of plant coiled-coil NLR-type pathogen receptors reveal essential roles of their N-terminal domain in oligomerization, networking, and immunity. PLOS Biology.

[ref-141] Xiao S, Ellwood S, Calis O, Patrick E, Li T, Coleman M, Turner JG (2001). Broad-spectrum mildew resistance in *Arabidopsis thaliana* mediated by RPW8. Science.

[ref-142] Xing H, Fu X, Yang C, Tang X, Guo L, Li C, Xu C, Luo K (2018). Genome-wide investigation of pentatricopeptide repeat gene family in poplar and their expression analysis in response to biotic and abiotic stresses. Scientific Reports.

[ref-143] Yang Y, Zhang Y, Li B, Yang X, Dong Y, Qiu D (2018). A *Verticillium dahliae* pectate lyase induces plant immune responses and contributes to virulence. Frontiers in Plant Science.

[ref-144] Yue JX, Meyers BC, Chen JQ, Tian D, Yang S (2012). Tracing the origin and evolutionary history of plant nucleotide-binding site-leucine-rich repeat (NBS-LRR) genes. New Phytologist.

[ref-145] Zhang C, Wang X, Zhang F, Dong L, Wu J, Cheng Q, Qi D, Yan X, Jiang L, Fan S, Li N, Li D, Xu P, Zhang S (2017). Phenylalanine ammonia-lyase2.1 contributes to the soybean response towards *Phytophthora sojae* infection. Scientific Reports.

[ref-146] Zhang R, Fan Z, Kasuga T (2011). Expression of cellobiose dehydrogenase from *Neurospora crassa* in *Pichia pastoris* and its purification and characterization. Protein Expression and Purification.

[ref-147] Zhang XC, Cannon SB, Stacey G (2009). Evolutionary genomics of LysM genes in land plants. BMC Evolutionary Biology.

[ref-148] Zhong Y, Cheng ZM (2016). A unique RPW8-encoding class of genes that originated in early land plants and evolved through domain fission, fusion, and duplication. Scientific Reports.

[ref-149] Zhu C, Wang Y, Li Y, Bhatti KH, Tian Y, Wu J (2011). Overexpression of a cotton cyclophilin gene (GhCyp1) in transgenic tobacco plants confers dual tolerance to salt stress and *Pseudomonas syringae* pv. *tabaci* infection. Plant Physiology and Biochemistry.

[ref-150] Zsigmond L, Rigó G, Szarka A, Székely G, Otvös K, Darula Z, Medzihradszky KF, Koncz C, Koncz Z, Szabados L (2008). Arabidopsis PPR40 connects abiotic stress responses to mitochondrial electron transport. Plant Physiology.

