# Supplementary material for: Molecular evidence of the avocado defense response to Fusarium kuroshium infection: a deep transcriptome analysis using RNA-Seq
Source: PeerJ. 2021 Apr 14;9:e11215. doi: 10.7717/peerj.11215 (PMC8052963; doi:10.7717/peerj.11215)
Supplement: Supplemental Information 1 — (A) Schematic representation of each of the metrics resulting from preprocessing, assembly, and sequence annotation. Wedge size reflects the number of unique sequences (paired-end reads or UniGenes) that were obtained after each step and that were processed and used to obtain the nonredundant sequence set that comprises the avocado coding sequence (CDS) collection. (B) Bar plot showing the number of Fusarium-like sequences identified in each of the libraries generated from the infected stem segments. Error bars show the standard deviation from biological replicates (n = 3). [file peerj-09-11215-s001.pdf]

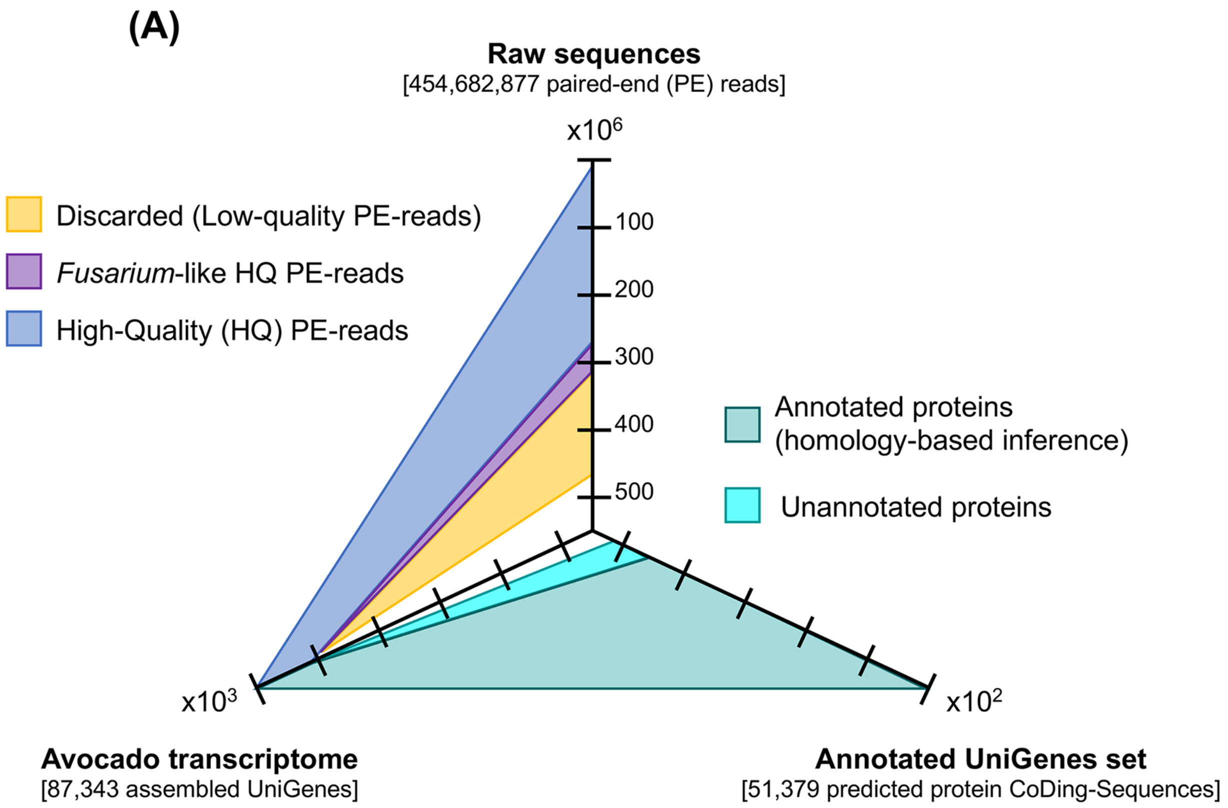

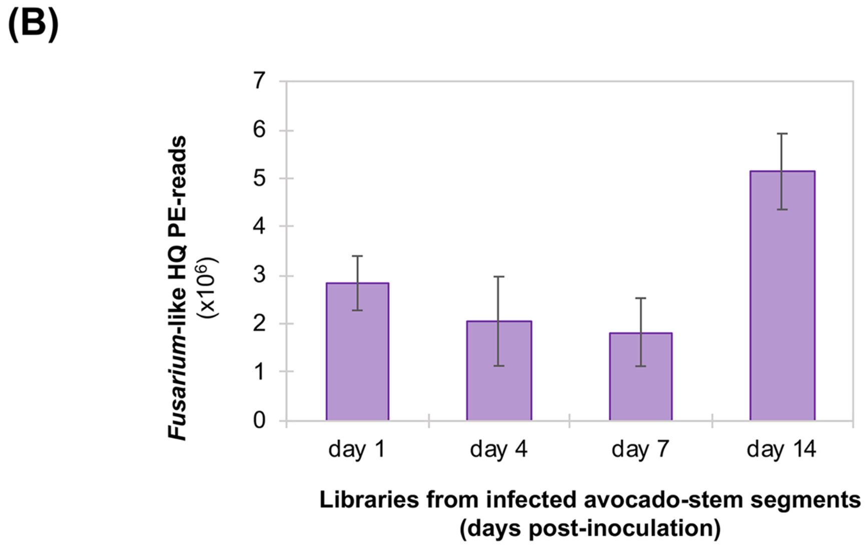

Figure S1. Quality check, data preprocessing, assembly, and avocado (*Persea americana*) transcriptome annotation. **(A)** Schematic representation of each of the metrics resulting from preprocessing, assembly, and sequence annotation. Wedge size reflects the number of unique sequences (paired-end reads or UniGenes) that were obtained after each step and that were processed and used to obtain the nonredundant sequence set that comprises the avocado coding sequence (CDS) collection. **(B)** Bar plot showing the number of *Fusarium*-like sequences identified in each of the libraries generated from the infected stem segments. Error bars show the standard deviation from biological replicates (n = 3).
