# Supplementary material for: Molecular evidence of the avocado defense response to Fusarium kuroshium infection: a deep transcriptome analysis using RNA-Seq
Source: PeerJ. 2021 Apr 14;9:e11215. doi: 10.7717/peerj.11215 (PMC8052963; doi:10.7717/peerj.11215)
Supplement: Supplemental Information 2 — The shared orthologs, as well as the unique genes identified between compared angiosperm plant species, were identified using OrthoMCL software (Li, Stoeckert & Roos, 2003). Each concentric circle corresponds to one compared species: [1] Amborella trichopoda, [2] Arabidopsis thaliana, [3] Musa acuminata subsp. malaccensis, [4] Persea americana, [5] Prunus persica, [6] Zea mays, [7] Solanum lycopersicum, and [8] Vitis vinifera. Cyan blocks mark the intersection of pairs (orthologs between two species) and show the species-specific genes from each of the species. The height of the purple bars in the outer layer (next to the last concentric circle) corresponds to the number of shared orthologs (x102). To generate this figure, we used the R package SuperExactTest (Wang, Zhao & Zhang, 2015). [file peerj-09-11215-s002.pdf]

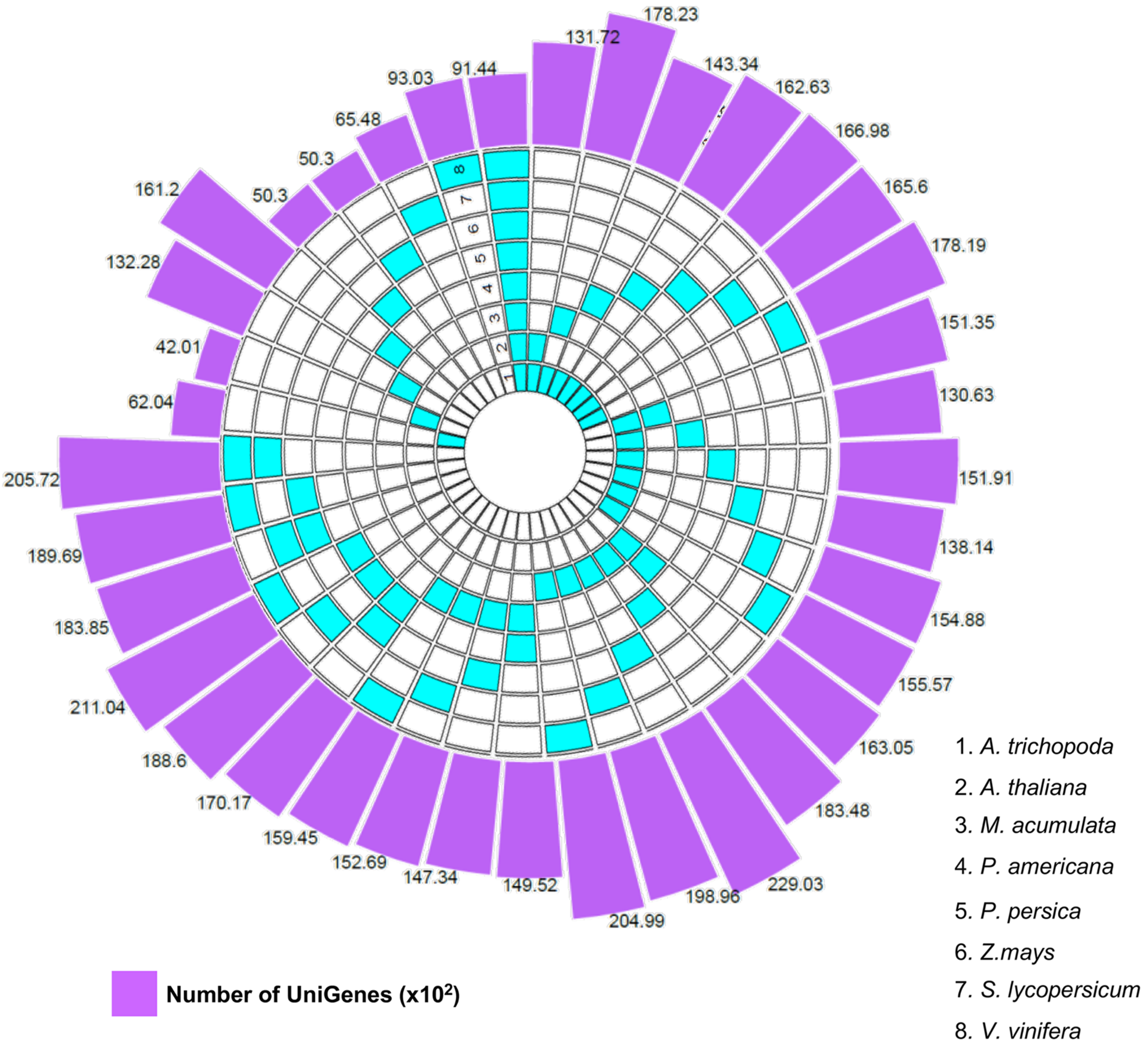

Figure S2. Orthologues detection in different plant species. The shared orthologs, as well as the unique genes identified between compared angiosperm plant species, were identified using OrthoMCL software (Li et al., 2003). Each concentric circle corresponds to one compared species: [1] *Amborella trichopoda*, [2] *Arabidopsis thaliana*, [3] *Musa acuminata* subsp. *malaccensis*, [4] *Persea americana*, [5] *Prunus persica*, [6] *Zea mays*, [7] *Solanum lycopersicum*, and [8] *Vitis vinifera*. Cyan blocks mark the intersection of pairs (orthologs between two species) and show the species-specific genes from each of the species. The height of the purple bars in the outer layer (next to the last concentric circle) corresponds to the number of shared orthologs ($x10^2$). To generate this figure, we used the R package SuperExactTest (Wang et al. 2015a).
