# Supplementary material for: Molecular evidence of the avocado defense response to Fusarium kuroshium infection: a deep transcriptome analysis using RNA-Seq
Source: PeerJ. 2021 Apr 14;9:e11215. doi: 10.7717/peerj.11215 (PMC8052963; doi:10.7717/peerj.11215)
Supplement: Supplemental Information 3 — Cluster analysis was performed with k-means methods (n = 6) using Genesis software (Sturn, Quackenbush & Trajanoski, 2002). The x-axis represents the days after inoculation (1-dpi, 4-dpi, 7-dpi, and 14-dpi, respectively). The y-axis represents the Log2 fold-change values calculated once infected and uninfected avocado stems-segments were compared. Gray lines, expression ofsingle genes trough the time; pink line, average of all genes in cluster. On the right side of k-means cluster graphs, a Heatmap of the differentially expressed UniGenes and contained in each cluster. Red, blue, and white indicate upregulation, downregulation, and no change, once infected and uninfected stems-segments were compared at 1, 4, 7 and 14 dpi. Each column represents the data from one day. The list of genes grouped in each cluster is shown in Table S6. [file peerj-09-11215-s003.pdf]

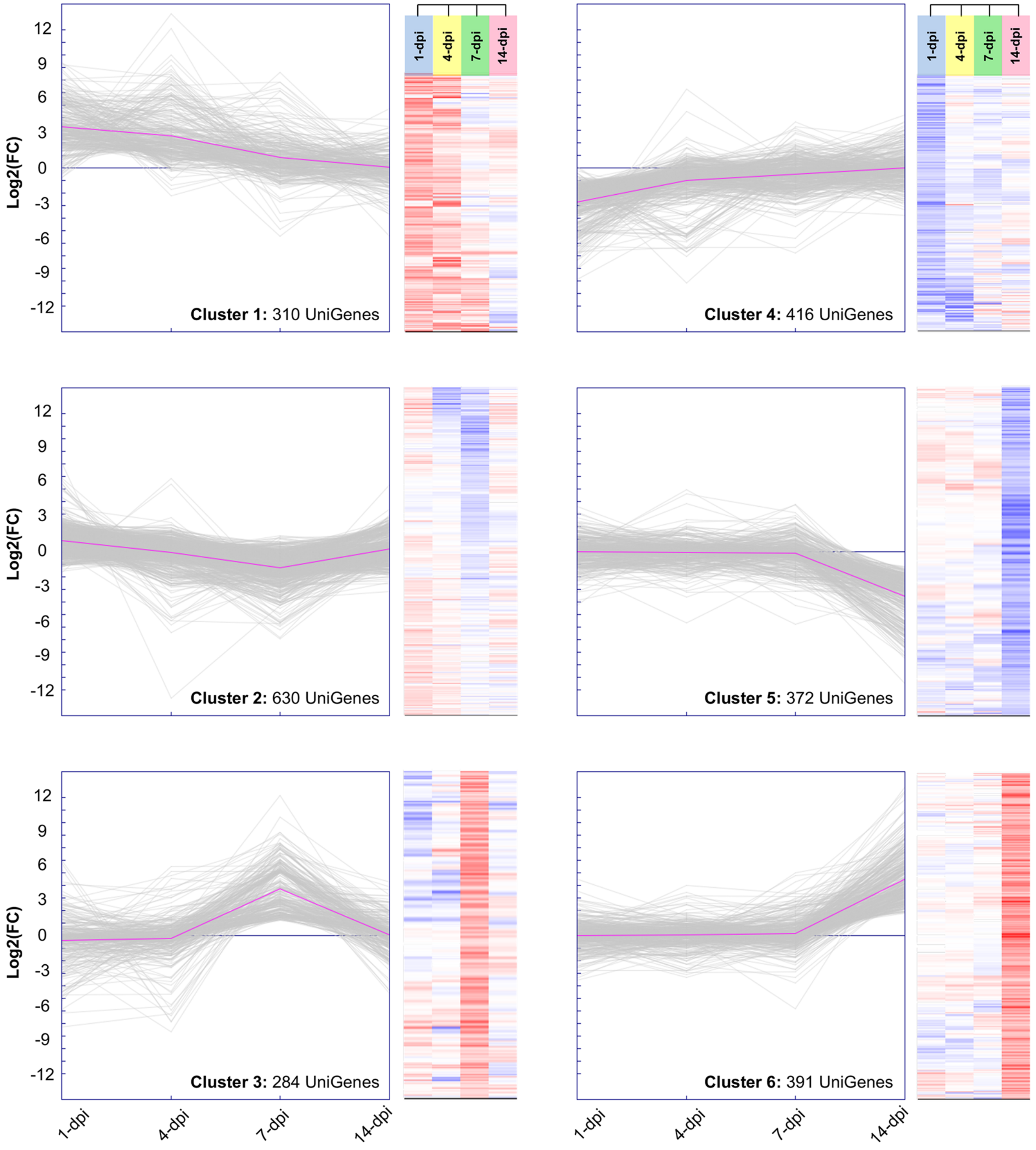

Figure S3. Expression profiles associated with the differentially expressed UniGenes involved in the responses to interaction with *F. kuroshium*. Cluster analysis was performed with k-means methods (n = 6) using Genesis software (Sturn et al. 2002). The x-axis represents the days after inoculation (1-dpi, 4-dpi, 7-dpi, and 14-dpi, respectively). The y-axis represents the Log_2 fold-change values calculated once infected and uninfected avocado stems-segments were compared. Gray lines, expression of single genes trough the time; pink line, average of all genes in cluster. On the right side of k-means cluster graphs, a Heat map of the differentially expressed UniGenes and contained in each cluster. Red, blue, and white indicate upregulation, downregulation, and no change, once infected and uninfected stems-segments were compared at 1, 4, 7 and 14 dpi. Each column represents the data from one day. The list of genes grouped in each cluster is shown in Table S6.
