# Supplementary material for: Molecular evidence of the avocado defense response to Fusarium kuroshium infection: a deep transcriptome analysis using RNA-Seq
Source: PeerJ. 2021 Apr 14;9:e11215. doi: 10.7717/peerj.11215 (PMC8052963; doi:10.7717/peerj.11215)
Supplement: Supplemental Information 4 — (A) The consensus sequence pattern of the MADA motif, which was identified in a total of 30 avocado CC-NLR proteins. Red boxes refer to residues that were highly conserved and previously reported in plant species (Adachi et al., 2019). (B) Three clearly distinguishable major groups (A, B and C) were identified after aligning and resolving their phylogenetic relationships based only on the CC domain, which contains a conserved array of amino acids known as the EDVID motif or the EDVID-corresponding area (Wróblewski et al., 2018). WebLogo (Crooks et al., 2004) with small-sample correction (Schneider et al., 1986) was used to generate the graphical representations of both domains (N-terminal domain CC-type and NBS, respectively). [file peerj-09-11215-s004.pdf]

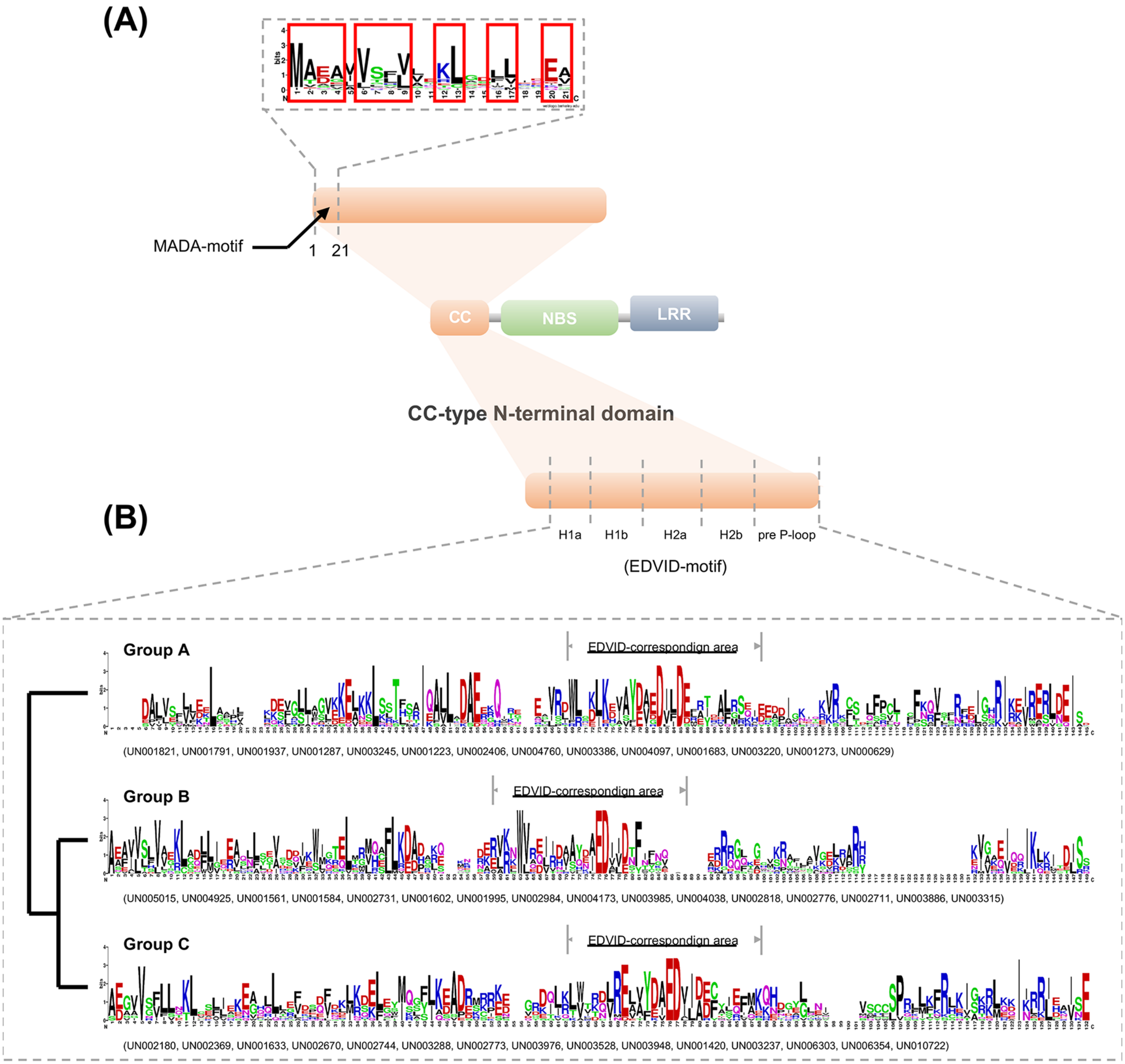


**Figure S4. Schematic representation of the motifs contained in the N-terminal domain of the avocado CC-type NLR proteins.** (A) The consensus sequence pattern of the MADA motif, which was identified in a total of 30 avocado CC-NLR proteins. Red boxes refer to residues that were highly conserved and previously reported in plant species (Adachi et al., 2019a). (B) Three clearly distinguishable major groups (A, B and C) were identified after aligning and resolving their phylogenetic relationships based only on the CC domain, which contains a conserved array of amino acids known as the EDVID motif or the EDVID-corresponding area (Wróblewski et al., 2018). WebLogo (Crooks et al., 2004) with small-sample correction (Schneider et al., 1986) was used to generate the graphical representations of both domains (N-terminal domain CC-type and NBS, respectively).
