# Supplementary material for: Molecular evidence of the avocado defense response to Fusarium kuroshium infection: a deep transcriptome analysis using RNA-Seq
Source: PeerJ. 2021 Apr 14;9:e11215. doi: 10.7717/peerj.11215 (PMC8052963; doi:10.7717/peerj.11215)
Supplement: Supplemental Information 5 — Schematic representation of the motifs contained in the NBS domain of the avocado CC-type NLR proteins. The consensus sequence of each of the highly conserved motifs in the NBS domain (P-loop, kinase, RNBS, GLPL, MHD; (Van Ghelder et al., 2019) was graphically displayed with WebLogo (Crooks et al., 2004) using small-sample correction (Schneider et al., 1986). [file peerj-09-11215-s005.pdf]

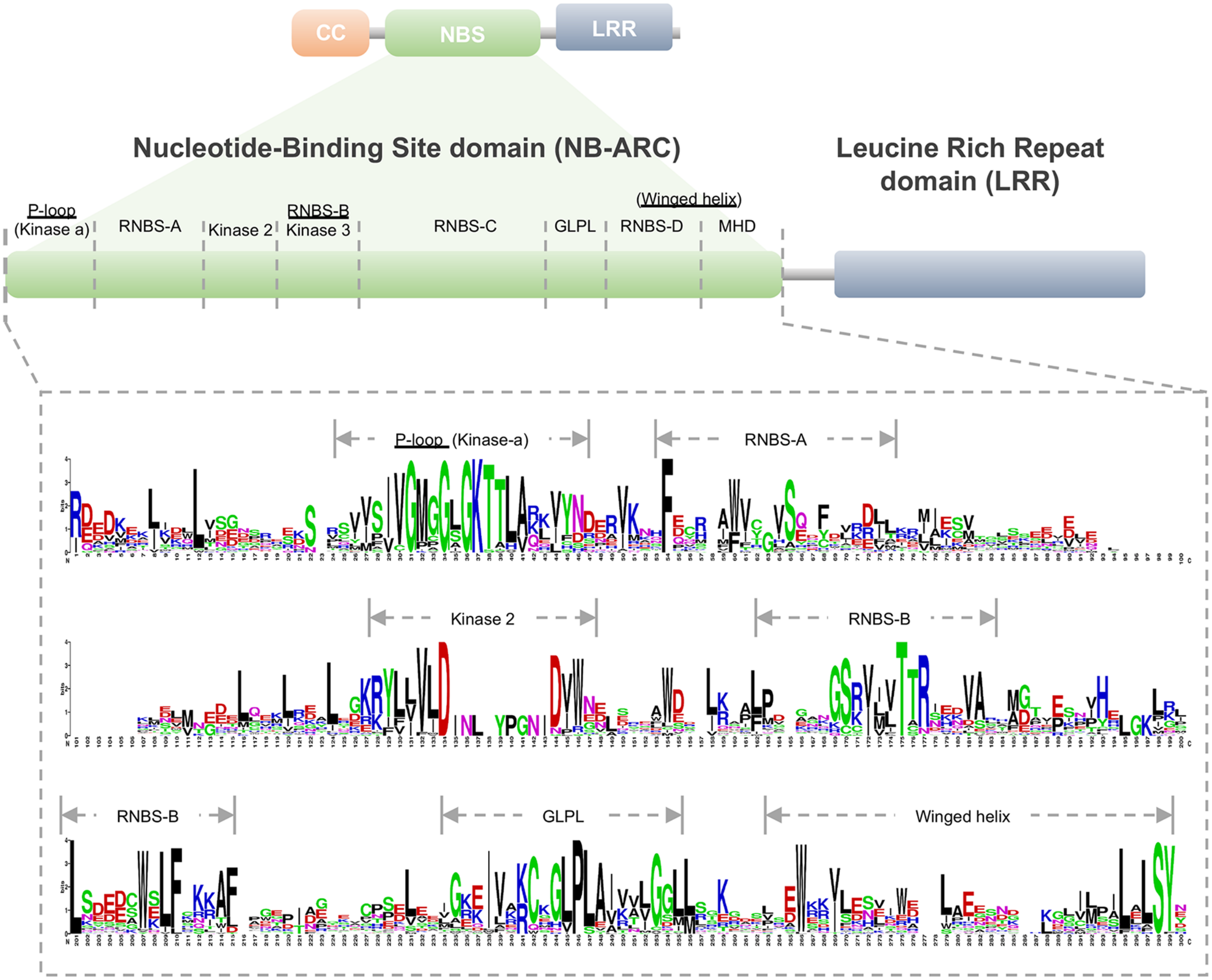

Figure S5. Schematic representation of the motifs contained in the NBS domain of the avocado CC-type NLR proteins. Schematic representation of the motifs contained in the NBS domain of the avocado CC-type NLR proteins. The consensus sequence of each of the highly conserved motifs in the NBS domain (P-loop, kinase, RNBS, GLPL, MHD; (Van Ghelder et al., 2019) was graphically displayed with WebLogo (Crooks et al., 2004) using small-sample correction (Schneider et al., 1986).
