# Supplementary material for: Molecular evidence of the avocado defense response to Fusarium kuroshium infection: a deep transcriptome analysis using RNA-Seq
Source: PeerJ. 2021 Apr 14;9:e11215. doi: 10.7717/peerj.11215 (PMC8052963; doi:10.7717/peerj.11215)
Supplement: Supplemental Information 6 — From left to right, the figure shows the side, top, and bottom views of the structure conformed by five monomers, all of them corresponding either to UN001791 (A) or UN003288 (B), respectively. These UniGenes encode avocado NLR receptors, both orthologs of the ZAR1 A. thaliana protein. To show these structures in their activated form, the ZAR1-RKS1-PBL21 resistosome [6j5t.pdb; (Wang et al., 2019a) was used as a template. In the modeling, no orthologs to RKS1 or PBL2 from avocado were included. The characteristic consensus sequence pattern of the MADA motif and its specific sequences in UN001791 and UN003288 are also shown (C) and are highlighted by red boxes in the putative resistosome structures. [file peerj-09-11215-s006.pdf]

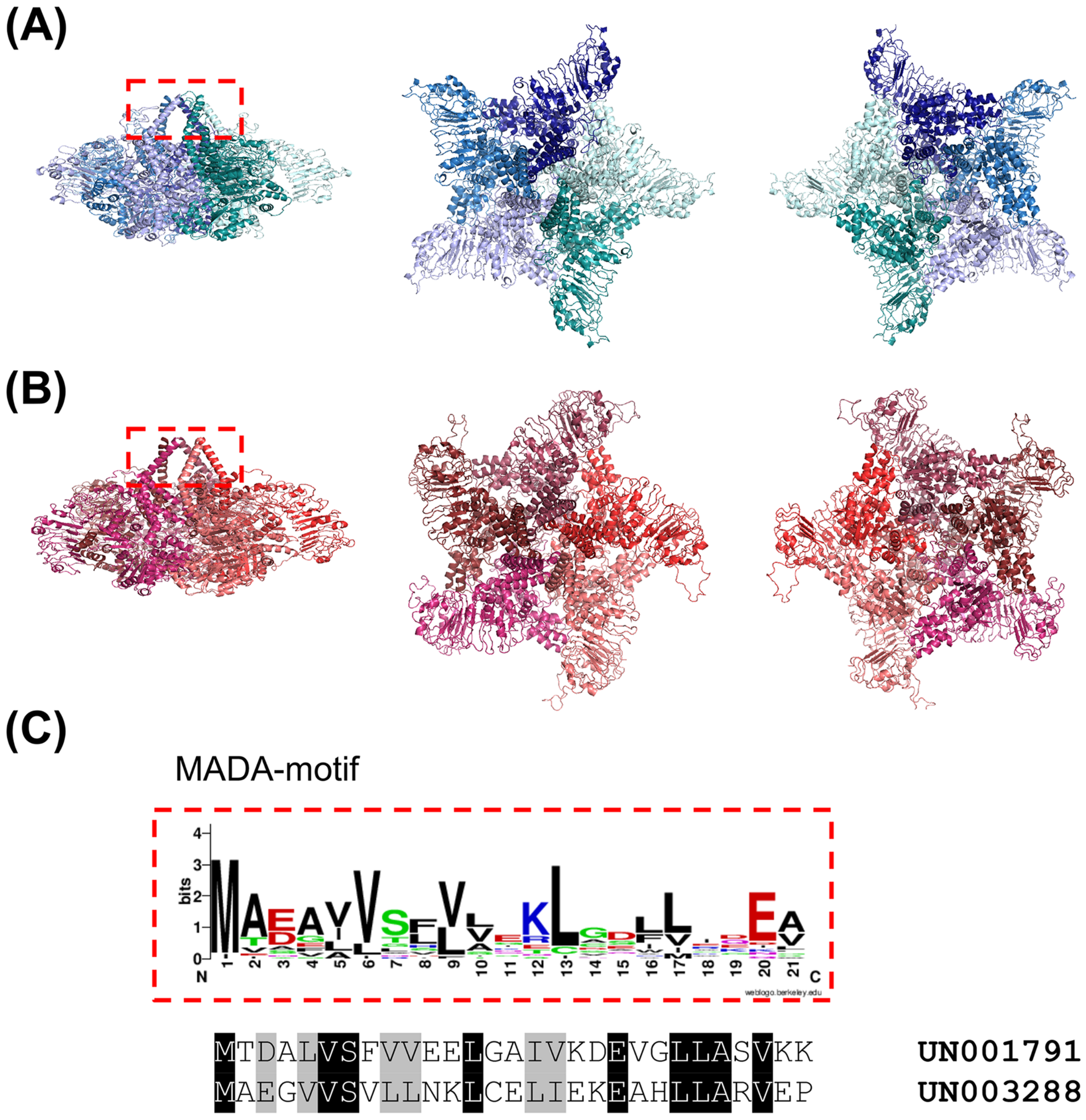

Figure S6. 3D structure models of a putative avocado resistosome. From left to right, the figure shows the side, top, and bottom views of the structure conformed by five monomers, all of them corresponding either to UN001791 **(A)** or UN003288 **(B)**, respectively. These UniGenes encode avocado NLR receptors, both orthologs of the ZAR1 *A. thaliana* protein. To show these structures in their activated form, the ZAR1-RKS1-PBL21 resistosome [6j5t.pdb; (Wang et al. 2019a) was used as a template. In the modeling, no orthologs to RKS1 or PBL2 from avocado were included. The characteristic consensus sequence pattern of the MADA motif and its specific sequences in UN001791 and UN003288 are also shown **(C)** and are highlighted by red boxes in the putative resistosome structures.
